# LncRNA AC010789.1 Promotes Colorectal Cancer Progression by Targeting MicroRNA-432-3p/ZEB1 Axis and the Wnt/β-Catenin Signaling Pathway

**DOI:** 10.3389/fcell.2020.565355

**Published:** 2020-10-15

**Authors:** Weili Duan, Xue Kong, Juan Li, Peilong Li, Yinghui Zhao, Tong Liu, Helen Barong Binang, Yunshan Wang, Lutao Du, Chuanxin Wang

**Affiliations:** ^1^Department of Clinical Laboratory, The Second Hospital, Cheeloo College of Medicine, Shandong University, Jinan, China; ^2^Tumor Marker Detection Engineering Technology Research Center of Shandong Province, Jinan, China; ^3^Tumor Marker Detection Engineering Laboratory of Shandong Province, Jinan, China; ^4^The Clinical Research Center of Shandong Province for Clinical Laboratory, Jinan, China

**Keywords:** colorectal cancer, AC010789.1, MicroRNA-432-3p, ZEB1, progression

## Abstract

Accumulating literatures have indicated that long non-coding RNAs (lncRNAs) are crucial molecules in tumor progression in various human cancers, including colorectal cancer (CRC). However, the clinical significance and regulatory mechanism of a vast majority of lncRNAs in CRC remain to be determined. The current study aimed to explore the function and molecular mechanism of lncRNA AC010789.1 in CRC progression. AC010789.1 found to be overexpressed in CRC tissues and cells. High expression of AC010789.1 was associated with lymph node metastasis and poor prognosis. Moreover, AC010789.1 silencing inhibited proliferation, migration, invasion and epithelial-mesenchymal transition (EMT) in vitro as well as tumorigenesis and metastasis in vivo. Mechanistically, we demonstrated that repression of AC010789.1 promoted miR-432-3p expression, and miR-432-3p directly binds to ZEB1. We then proved the anti-tumor role of miR-432-3p in CRC, showing that the inhibitory effect of AC010789.1 knockdown on CRC cells was achieved by the upregulation of miR-432-3p but downregulation of ZEB1. We also established that silencing AC010789.1 suppressed the Wnt/β-catenin signaling pathway. However, this inhibitory effect was partially counteracted by inhibition of miR-432-3p. In summary, these results reveal that silencing AC010789.1 suppresses CRC progression via miR-432-3p-mediated ZEB1 downregulation and suppression of the Wnt/β-catenin signaling pathway, highlighting a potentially promising strategy for CRC treatment.

## Introduction

Colorectal cancer (CRC) is one of the most common malignant tumors and the leading cause of cancer deaths worldwide (Siegel et al., 2019). Tumor progression and metastasis are the leading causes of deaths due to CRC. Despite the significant progress made in diagnostic methods and improved treatment strategies, the prognosis of CRC patients remains poor, especially in those with advanced CRC (Yang and Weinberg, 2008; Jung et al., 2020; Mauri et al., 2020). This makes the understanding of the process of metastasis critical to improve the prognosis of CRC. Tumor metastasis is a complex process; one of the significant developmental program is EMT, which permits invasion and migration of cancer cells (Aiello et al., 2018; Pastushenko et al., 2018), and is associated with poor prognosis (Yang and Weinberg, 2008; Singh and Settleman, 2010; De Craene and Berx, 2013; Shang et al., 2019). Tumor progression is regulated by several biomolecules, whose mechanisms are mostly unknown (Zhang C. et al., 2020). Therefore, there is an urgent need to clarify the mechanisms underlying CRC progression.

Non-coding RNAs (ncRNAs) have attracted considerable research interest in recent years as functional data suggest that they play essential roles in multiple human tumors (Dragomir et al., 2020; Skrzypek and Majka, 2020). Among these ncRNAs, microRNAs (miRNAs) and long non-coding RNAs (lncRNAs) have appealed to a large group of researchers. miRNAs impact oncogenes and tumor suppressor genes stability and the rate of translation by binding to complementary sites within target RNAs (Vos et al., 2019; Petri and Klinge, 2020). LncRNAs are now described to have many functions in promoting and maintaining tumor initiation and progression (Kim and Croce, 2018; Hu et al., 2020). Intriguingly, several lines of evidence demonstrated a brand new lncRNAs regulatory mechanism in which lncRNAs may serve as competing endogenous RNAs (ceRNAs) and crosstalk with mRNAs (Qi et al., 2015; Li et al., 2020). A ceRNA is a transcript targeted by a miRNA that, in doing so, sequester miRNAs with greater efficacy, thereby upregulating miRNA target gene expression (Thomson and Dinger, 2016; Zhang M. et al., 2020). Song et al. found that lncRNA-KRTAP5-AS1 and lncRNA-TUBB2A could act as ceRNAs to affect the function of Claudin-4 (Song et al., 2017). Liang et al. identified that LncRNA BCRT1 upregulated PTBP3 expression by competitively binding the miR-1303 and accelerated breast cancer progression (Liang et al., 2020). Likewise, FN1 was a functional ceRNA for miR-200c during TGF-β-induced EMT (Liu et al., 2019). Up to now, many molecular mechanisms of metastasis have been investigated. However, the role of potential networks between ncRNAs and mRNA in CRC has not been fully elucidated.

The current study aimed to explore the possible function of AC010789.1 in CRC progression and metastasis. We identified that AC010789.1 was upregulated in CRC tissues and associated with poor clinical prognosis. Besides, silencing AC010789.1 inhibited CRC migration, invasion, and EMT in vivo and in vitro. Mechanistically, AC010789.1 was shown to promote CRC progression through sponging miR-432-3p, leading to the upregulation of ZEB1 and activation of the Wnt/β-catenin signaling pathway. Therefore, in this study, we obtained insights into the function and mechanisms underlying AC010789.1 regulation of CRC progression and metastasis.

## Materials and Methods

### Patients and Specimens

In total, 86 pairs of freshly collected tumor tissue samples, and adjacent nontumor tissues were randomly obtained from CRC patients without preoperative treatment at The Second Hospital of Shandong University from January 2015 to August 2018. All tissue samples were immediately frozen in liquid nitrogen after surgical resection from CRC patients and stored at −80°C until used for RNA extraction. The pieces of information of CRC patients are described in Supplementary Table 1. Informed consent was obtained from all patients. Permission for the use of human samples in this study was granted by the Committee for Ethical Review of Research involving Human Subjects of The Second Hospital, Cheeloo College of Medicine, Shandong University.

### Cell Lines

The immortalized colon epithelial cell line FHC and CRC cell lines (HCT116, SW1116, SW480, HT29, and DLD1) were obtained from the Chinese Academy of Sciences Cell Bank. Cell lines were cultured with DMEM supplemented with 10% fetal bovine serum and penicillin-streptomycin at 37°C in 5% CO_2_ and 95% air.

### Luciferase Reporter Assay

For luciferase reporter assays, we constructed wild-type AC010789.1 reporter plasmid (AC010789.1-WT), mutated-type AC010789.1 reporter plasmid (AC010789.1-MUT), wild-type ZEB1-3′ untranslated regions (UTR) reporter plasmid (ZEB1-WT) and mutated-type ZEB1-3′ UTR reporter plasmid (ZEB1-MUT) with pmirGLO promoter vector. The pmirGLO vector with either wild-type fragments or mutation fragments was co-transfected with mimic NC or miR-432-3p mimic by Lipofectamine 2000 according to the manufacturer’s guidelines. 48 h after transfection, the luciferase signal was detected using the Dual-Luciferase^®^ Reporter Assay System (Promega, United States) according to the manufacturer’s instructions. The experiment was conducted three times independently.

### *In situ* Hybridization

The CRC Tissue Microarray (TMA) experiments were conducted by Shanghai Outdo Biotech Co., Ltd. (HColA180Su17). The CRC TMA containing 101 CRC patient cases, 79 of which had adjacent normal tissues from the same patient. All of these patients have detailed clinical information, including age, gender, TNM stage and so on (Supplementary Table 2). The *In situ* Hybridization (ISH) results were scored based on two parameters: (I) staining intensity score as 0 (negative), 1 (1+) or 2 (2+); (II) staining positive rate score as 0 (negative), 1 (1–25%), 2 (26–50%), 3 (51–75%), or 4 (7–100%). The product of “staining intensity score” and “staining positive rate score” was used as the total score for grouping, ≤4 was regarded as the probe low-expression group, and >4 was regarded as the probe high-expression group. All experiments were approved by the ethics committee of Shanghai Outdo Biotech Company.

### RNA Extraction and Quantitative Real-Time Polymerase Chain Reaction (qRT-PCR) Analysis

The total RNA of tissues and CRC cell lines were extracted using TRIzol reagent (Invitrogen, Carlsbad, CA, United States) and Fastagen (Feijie Biotech, Shanghai, China) respectively. The RNA concentration and purity were measured by Nanodrop 2000 (Thermo Scientific). The RNA extracted was reverse transcribed by the PrimeScript^TM^ RT reagent kit (Takara, Dalian, China). qRT-PCR was carried out using the SYBR Premix Ex Taq ^TM^ (Takara, Dalian, China); β-actin was used as the endogenous control to normalize lncRNA and mRNA expressions. For miRNA expression analysis, 1 μg of total RNA was reverse transcribed into cDNA and qRT-PCR was performed in the CFX-96 real-time PCR System (BIO-RAD, United States) with All-in-One^TM^ miRNA qRT-PCR Detection Kit (GeneCopoeia, Carlsbad, CA, United States); the results were normalized to U6 expression. The specific Primers used are listed in Supplementary Table 3. All reactions were carried out in triplicate. The relative expressions of lncRNAs, mRNA, and miRNA from tissue samples and cell lines were calculated using the 2^–ΔΔ*ct*^ method.

### RNA Oligoribonucleotides and Cell Transfections

The short interfering RNA (siRNA) specific for AC010789.1, miR-432-3p mimic, mimic negative control (NC), miR-432-3p inhibitor, and inhibitor NC oligonucleotides were obtained from GenePharma (Shanghai, China). Lipofectamine 2000 (Invitrogen) was used for cell transfection according to their manufacturer’s instructions.

### Generation of Stable Cell Lines

To establish stable AC010789.1 knockdown cells, a lentivirus vector encoding specific shRNA sequences or the negative control vector was constructed, which can express the green fluorescent protein (GFP) and puromycin resistance gene. After the transfection of the lentivirus vector, the cells were then subjected to drug selection (2.5 μg/ml puromycin). The expression of GFP was observed under a fluorescence microscope to confirm the transfection efficiency, and efficiency of the knockdown was verified by qRT-PCR.

### Overexpression Plasmid Construction

To construct a plasmid overexpressing AC010789.1, the full-length human AC010789.1 sequence was synthesized and subcloned into the pEX-3 vector (GenePharma, Shanghai, China). The pENTER-ZEB1 plasmid for upregulation of the ZEB1 gene was obtained from Vigene BioSciences. Besides, the pENTER-ZEB1 plasmid does not contain the 3′UTR region of ZEB1, which is the site where miR-432-3p binds. Lipofectamine 2000 (Invitrogen) was used for cell transfection of plasmid according to the manufacturer’s instructions.

### Real-Time Cell Analysis

Each well of *E*-plates was filled with 50 μl of culture media, and the background impedance measurement was performed within 10 min. Cell suspensions with cell density 5 × 10^3^ cells/well were seeded into the *E*-plate. Then, the *E*-plate was incubated for 30 min at room temperature to allow for an initial cells’ adhesion at the bottom of each well. Then the plates were plated in 37°C incubators. The Real-Time Cell Analysis (RTCA) software was used for cell index values analysis.

### Colony Formation Assay

Cells transfected with si-AC010789.1 for 48 h were trypsinized into a single-cell suspension. Then cells were seeded (1000 cells per well) in six-well plates. After 14 days, the plates were gently washed with phosphate-buffered saline (PBS) and fixed with 4% paraformaldehyde for 30 min, then stained using crystal violet. Colonies with over 50 cells were manually counted.

### Transwell Migration and Invasion Assays

The in vitro cell migration assay was performed using transwell chambers (8 μm pore size; Costar), and transwell invasion assay was conducted using the transwell chambers (8 μm pore size; Costar) and Matrigel (BD Biosciences, San Jose, CA, United States), according to the manufacturer’s instructions. For migration and invasion assays, media containing 20% FBS in the lower chamber served as a chemoattractant. After 24 or 48 h, the nonmigrating or noninvasive cells were removed from the upper face of the filters using cotton swabs. The migrated and invaded cells located on the lower side of the chamber were fixed with 4% paraformaldehyde and stained using crystal violet, air dried, photographed and counted. The number of migratory and invasive cells were counted in 5 randomly selected visual fields from the central and peripheral portions of the filter.

### Immunofluorescence Assay

HCT116 cells with AC010789.1 knockdown were cultured and fixed on 12 mm × 12 mm glass slides. After incubation with antibodies specific for E-cadherin and Vimentin, the slides were incubated with a fluorescent secondary antibody for 2 h at room temperature in the dark. Finally, the slides were mounted by adding DAPI-Fluoromount-G (Southern Biotech, SBA, Birmingham, AL). Images were captured with a microscope (Zeiss, Germany) and quantified using ImageJ software.

### Western Blot Assay

Total cell lysates were obtained using the RIPA buffer (Sigma-Aldrich) and quantified with the BCA Protein Quantification Kit (Vazyme Biotech Co. Ltd., Nanjing, China). Proteins were separated by SDS-polyacrylamide gel electrophoresis (SDS–PAGE) and transferred to Immobilon-P PVDF membrane (Millipore, United States). After being blocked with 5% nonfat milk in TBS-Tween 20 for 2 h at room temperature, membranes were incubated at 4°C overnight with primary specific antibodies to β-actin (cell signaling technology#3700S, 1:1000), β-catenin (cell signaling technology#8480T, 1:1000), vimentin (cell signaling technology#D21H3, 1:1000), E-cadherin (cell signaling technology#3195S, 1:1000), Cyclin D1 (cell signaling technology#2978S, 1:1000) C-myc (Abcam#AB32072, 1:1000), Lamin B1 (Proteintech#12987-1-AP, 1:1000), TCF8/ZEB1 (cell signaling technology#3396, 1:1000). After incubation with respective horseradish peroxidase-conjugated secondary antibodies, the blots were detected using the High-sensitivity ECL Chemiluminescence Detection Kit (Vazyme Biotech Co. Ltd, Nanjing, China) and quantified using ImageJ software.

### RNA Sequencing Analysis

Total RNA was extracted from HCT116 cells with AC010789.1 knockdown using the RNeasy mini kit (Qiagen, Germany). TruSeq^TM^ RNA Sample Preparation Kit (Illumina, United States) was used to synthesize the paired-end libraries according to the TruSeq^TM^ RNA Sample Preparation Guide. The mRNA expression profiles of the treated HCT116 cells were determined using the Illumina NovaSeq 6000 (Illumina, United States) following the manufacturer’s instructions. The library construction and sequencing were performed at the Shanghai Sinomics Corporation. Cuffdiff was used to evaluate differentially expressed genes. The differentially expressed mRNAs were selected using the following filter criteria: *P* < 0.05 and log2| fold change| >1.

### Animal Experiments

For the in vivo tumor formation assay, HCT116 cells stably transfected with sh-AC010789.1 and shRNA-NC were subcutaneously injected into the right armpit region of 12 five-week-old male BALB/c nude mice which were randomly divided into two groups (*n* = 6 for each group). The tumor volume was measured every 4 days. Twenty days after injection, the animals were sacrificed, and the xenograft tumors were isolated and processed for further assays.

For metastasis experiments, 1 × 10^6^ HCT116 cells stably transfected with sh-AC010789.1 and shRNA-NC in 0.1 ml PBS were injected into the tail vein of 12 five-week-old male BALB/c nude mice which were randomly divided into two groups (*n* = 6 for each group). After 5 weeks of injection, lung tissues were isolated from the mice, and tissue sections were stained with hematoxylin and eosin. The numbers of metastatic lung nodules were counted in five random sections per lung, and the operators and investigators were blinded to the group allocation. All animal studies were performed with approval from the Institutional Animal Care and Use Committee of The Second Hospital, Cheeloo College of Medicine, Shandong University.

### TCGA Gene Expression Datasets Analyses

The RNA sequencing data of 647 CRC tissues and 51 healthy tissues, including clinicopathological information, were downloaded from The Cancer Genome Atlas (TCGA) database. The clinical and demographic characteristics for TCGA CRC are shown in Supplementary Table 4. The data were preprocessed by using R software and packages. Differently expressed lncRNAs were detected with the “DESeq2” package in R software. LncRNAs with adjusted *P*-values < 0.05 and log2| fold change| >3 were considered statistically significant. To identify prognostic lncRNAs, the univariate Cox regression and Kaplan-Meier analysis were applied to analyze the survival-associated lncRNAs, *P* < 0.05 was considered statistically significant. This study met the publication guidelines and data access policies provided by TCGA^1^.

### Statistical Analysis

All statistical analyses were assessed using SPSS 19.0 (SPSS, Chicago, IL, United States) or GraphPad Prism5 (GraphPad Prism, Inc., La Jolla, CA, United States). The data between two groups were compared using the student’s *t*-test and one-way analysis of variance (ANOVA) were used to compare the variance of multiple groups. The nonparametric Mann-Whitney test was performed to compare the correlation between AC010789.1 level and clinical parameters. Correlation between AC010789.1, miR-432-3p, and ZEB1 were analyzed using Spearman correlation tests. Survival curves were estimated with the Kaplan–Meier method. All experiments were performed in triplicate and data are presented as mean ± SEM from triplicate independent experiments. *P* < 0.05 was considered statistically significant.

## Results

### AC010789.1 Is Upregulated in CRC and Associated With Poor Prognosis

**FIGURE 1:**
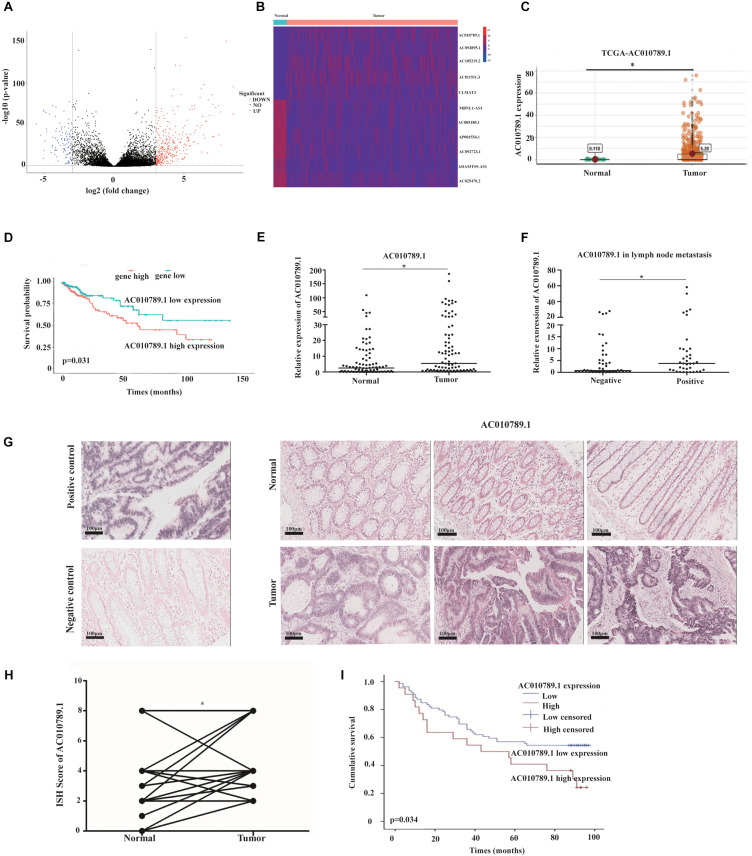
AC010789.1 is upregulated in CRC tissues, and its overexpression correlates with poor outcome of CRC. **(A)** Volcano plot of expression changes greater than triple of lncRNAs in CRC and normal tissues. (criteria: *P* < 0.05 and | log2FC| >3). **(B)** Heat map of lncRNAs that were abnormally expressed and associated with prognosis in CRC (criteria: *P* < 0.05). **(C)** AC010789.1 expression level in CRC tissues (*n* = 647) and normal tissues (*n* = 51) analyzed using the TCGA database. **(D)** Kaplan-Meier analysis of the overall survival of CRC patients with high and low levels of ACO10789.1 using the TCGA database. **(E)** Expression level of AC010789.1 in an additional CRC tissues (*n* = 86) and normal tissues (*n* = 86) were detected by qRT-PCR. **(F)** Relative expression level of AC010789.1 in CRC with lymph node metastasis (*n* = 37) and non-lymph node metastasis (*n* = 49). **(G)** ISH analysis of AC010789.1 expression in CRC tissues (*n* = 76) and normal tissues (*n* = 76) (Positive control, U6; negative control, scrambled lncRNA control), Scale bar: 100 μm. **(H)** ISH analysis of AC010789.1 expression in CRC and paired normal tissues. **(I)** Kaplan-Meier analysis for overall survival of CRC patients with low and high AC010789.1 expression (*P* = 0.034), *P < 0.05.

The RNA sequencing data of 647 CRC tissues and 51 normal tissues, including clinicopathological information (Supplementary Table 4), were downloaded from TCGA^2^ database. An adjusted *P* < 0.05 and the absolute log2 fold change (log2FC) >3 was considered statistically significant. A total of 435 differentially expressed lncRNAs were identified in patients with CRC (Figure 1A). Among them, 11 lncRNAs were associated with overall survival through univariate Cox regression and Kaplan-Meier analysis (*P* < 0.05) (Figure 1B). We focused on the up-regulated lncRNAs because they are more likely to be used as early diagnostic markers or therapeutic targets, compared to the downregulated lncRNAs. In particular, we selected AC010789.1 for further analysis. AC010789.1 was significantly elevated in CRC tissues compared to normal tissues (Figure 1C), and patients with high AC010789.1 expression had shorter survival times in the TCGA database (Figure 1D). To validate TCGA database findings, we examined the expression level of AC010789.1 in 86 CRC tissues and adjacent normal tissues. We demonstrated that AC010789.1 was upregulated (Figure 1E). Correlation with clinical data revealed that AC010789.1 expression level significantly correlated with higher rates of lymph node metastasis (*P* = 0.02) (Figure 1F and Supplementary Table 5). Moreover, AC010789.1 expression was significantly upregulated in CRC cell lines compared to FHC (Supplementary Figure 1A).

To further test whether AC010789.1 expression correlated with poor outcome of CRC, the expression level of AC010789.1 was measured by ISH in CRC patients with different clinicopathological features. The results showed that AC010789.1 expression level was higher in CRC tissues compared with paired normal tissues (Figures 1G,H and Supplementary Figure 1B). Moreover, Kaplan-Meier survival analysis revealed that patients with high AC010789.1 level had significantly lower overall survival (*P* = 0.034) (Figure 1I). Accordingly, these results strongly indicated that AC010789.1 was significantly overexpressed in CRC tissues and might be an essential factor for predicting prognosis in CRC patients.

### Knockdown of AC010789.1 Suppresses Proliferation, Migration, Invasion, and EMT of CRC Cells

**FIGURE 2:**
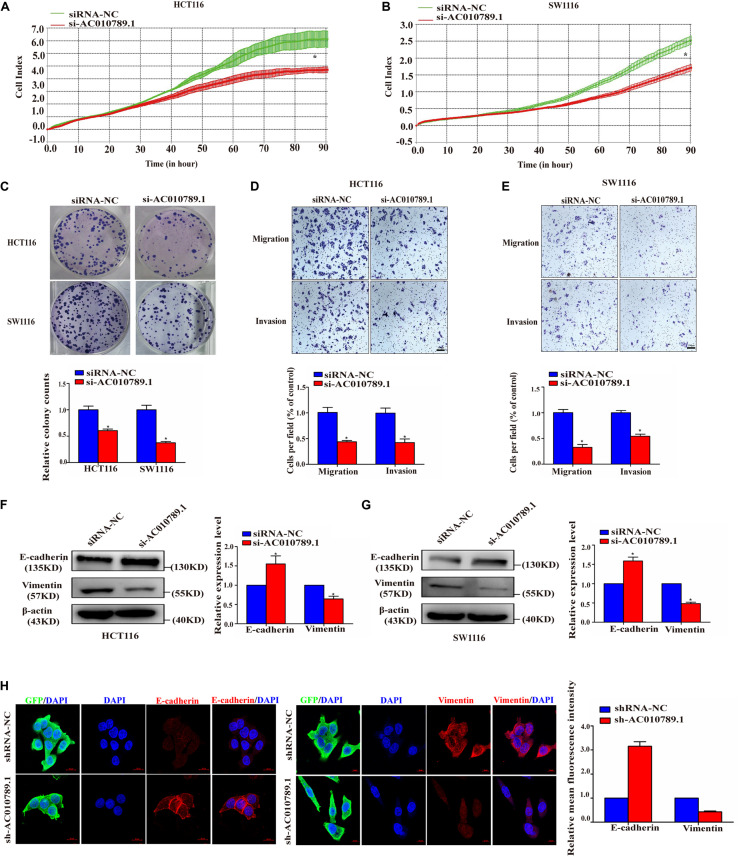
Silencing AC010789.1 inhibits proliferation, migration, invasion, and EMT of CRC cells in vitro. **(A,B)** The si-AC010789.1 mediated effect on proliferation was monitored by measuring cell impedance using the RTCA instrument. **(C)** Colony formation assay in HCT116 and SW1116 cells with AC010789.1 knockdown. **(D,E)** Transwell assay for investigating migration and invasion change in HCT116 and SW1116 cells after AC010789.1 knockdown, Scale bar: 100 μm. **(F,G)** The relative expression levels of E-cadherin and vimentin were determined by western blot in HCT116 and SW1116 cells with AC010789.1 knockdown. **(H)** The shRNA-NC and sh-AC010789.1 stable HCT116 cells were stained with antibodies to E-cadherin (red), vimentin (red) and GFP (green) and then stained with DAPI to detect nuclei (blue). Scale bar: 10 μm. Data are representative of three independent experiments and are presented as mean ± SEM. *P*-values were determined by two-tailed student’s *t*-test (*P < 0.05).

To better understand the biological effects of AC010789.1 on CRC development, loss-of-function experiments were conducted. Sine AC010789.1 has the highest expression in HCT116 and SW1116 cells, we used siRNAs to silence AC010789.1 expression in these two cell lines (Supplementary Figure 1A). The knockdown efficiency of AC010789.1 in HCT116 and SW1116 cells were verified by qRT-PCR and we selected siAC010789.1-2 for further study (abbreviated as si-AC010789.1) (Supplementary Figure 2A). Subsequently, RTCA assays were performed, and results showed that the downregulation of AC010789.1 resulted in decreased cell proliferation (Figures 2A,B). Colony formation assays demonstrated that the silencing of AC010789.1 decreased the numbers of colonies (Figure 2C). Next, it was clear that the silencing of AC010789.1 significantly reduced the migratory and invasive capabilities of HCT116 and SW1116 cells (Figures 2D,E). Also, Western blot analysis identified increased epithelial maker E-cadherin expression, and decreased mesenchymal maker vimentin expression after AC010789.1 downregulation (Figures 2F,G). The qRT-PCR analysis showed similar trends of the expressions of E-cadherin and vimentin (Supplementary Figures 2B,C). To further investigate the role of AC010789.1 in regulating EMT of CRC cell, HCT116-shAC010789.1 and SW1116-shAC010789.1 stable cell lines were, respectively, established (Supplementary Figures 2D–F). Immunofluorescence staining revealed an increase in E-cadherin expression as well as the loss of vimentin expression in HCT116-shAC010789.1 stable cells (Figure 2H). Taken together, these experiments revealed essential functions of AC010789.1 in CRC cell proliferation, migration, invasion and EMT.

### AC010789.1 Silencing Inhibits Tumorigenicity and Metastasis of CRC Cells *in vivo*

**FIGURE 3:**
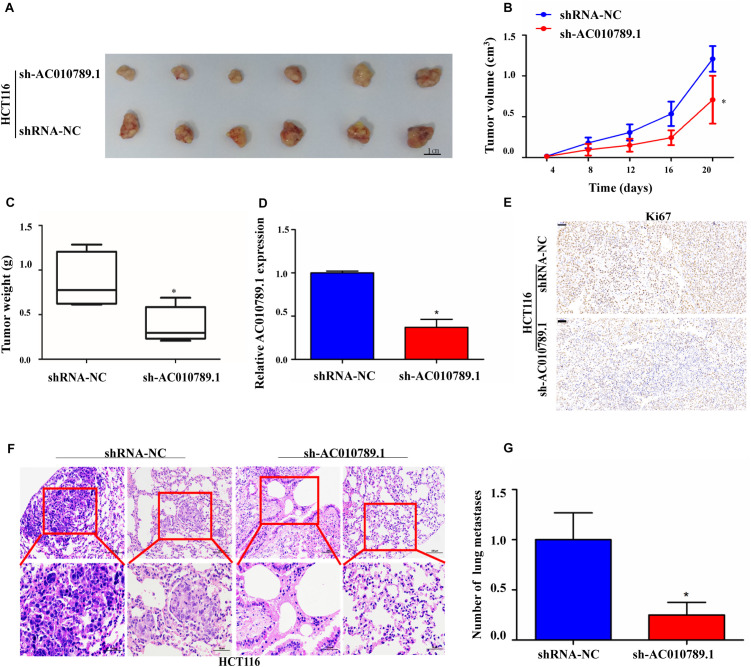
Silencing AC010789.1 inhibits tumor growth and metastasis in vivo. **(A)** The subcutaneous xenograft tumors of HCT116 cells isolated from nude mice; tumors were photographed from each group (*n* = 6), Scale bar: 1 cm. **(B)** The tumor growth curves of in vivo tumor volumes (*n* = 6/group). **(C)** Tumor weights of each group (*n* = 6) were also measured. **(D)** The qRT-PCR was performed to detect the expression level of AC010789.1 in the tumors (*n* = 6/group). **(E)** Representative images of immunohistochemistry stained for Ki67 using the tumor section, Scale bar: 50 μm. **(F)** The microscopic images of lung tissue sections stained by hematoxylin and eosin. **(G)** The number of metastatic nodules in the lung were counted. Data are presented as mean ± SEM, *n* = 6 for each group. *P*-values were determined by two-tailed student’s *t*-test (*P < 0.05).

To further explore the role of AC010789.1 in tumor development and progression in vivo, we established a nude mice xenograft model by implanting HCT116 sh-AC010789.1 or shRNA-NC stable cells. The results showed that the tumor volumes and the mean weights were significantly decreased in the sh-AC010789.1 group compared with those of the shRNA-NC group (Figures 3A–C). We performed qRT-PCR to evaluate AC010789.1 expression in xenografted tumor tissues. As expected, tumors formed from sh-AC010789.1 stable cells exhibited decreased AC010789.1 expression (Figure 3D). Moreover, tumor cell proliferation was assessed using proliferation-related nuclear antigen ki67 immunoreactivity. As shown in Figure 3E, the downregulation of AC010789.1 inhibited tumor cell proliferation. Then, we investigated the effect of AC010789.1 on CRC metastases by a tail vein injection model. Nude mice were injected via tail vein with 1 × 10^6^ cells, 5 weeks after injection, we found that sh-AC010789.1 stable cells produced significantly weaker metastasis as compared with shRNA-NC cells (Figures 3F,G). These data showed that AC010789.1 silencing inhibited tumor proliferation and metastasis in vivo.

### MiR-432-3p Directly Binds to AC010789.1 Producing an Anti-tumor Effect in CRC Cells

**FIGURE 4:**
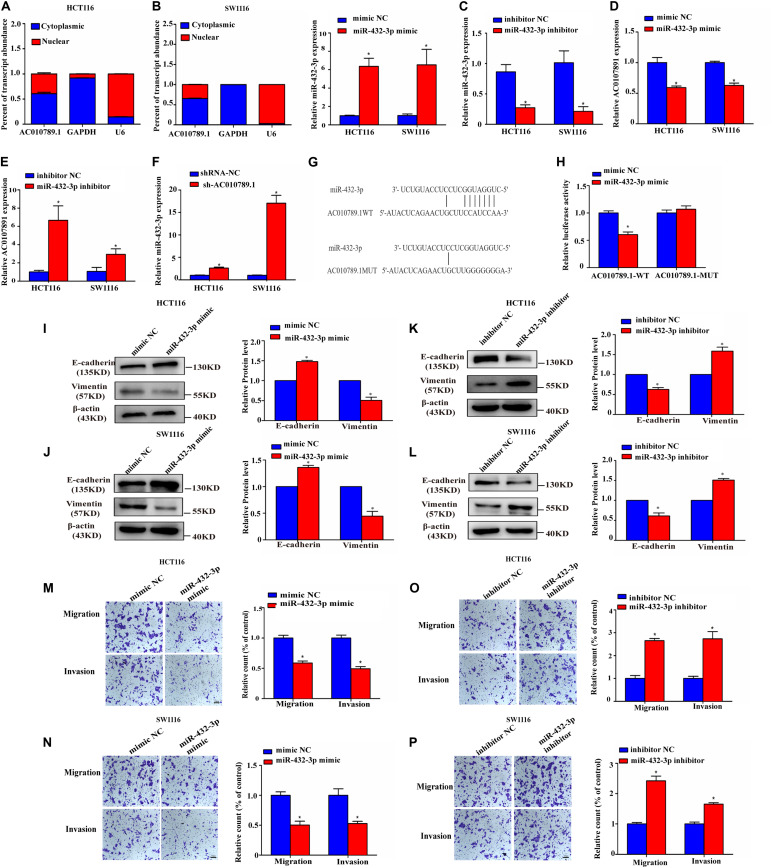
MiR-432-3p directly binds to AC010789.1, producing an anti-tumor effect in CRC cells. **(A)** Level of AC010789.1 in the nuclear and cytoplasmic fractions of HCT116 and SW1116 cells. **(B)** Relative expression of miR-432-3p in HCT116 and SW1116 cells transfected with mimic NC or miR-432-3p mimic. **(C)** Relative expression of miR-432-3p in HCT116 and SW1116 cells transfected with inhibitor NC or miR-432-3p inhibitor. **(D)** Relative expression of AC010789.1 in HCT116 and SW1116 cells transfected with mimic NC or miR-432-3p mimic. **(E)** Relative expression of AC010789.1 in HCT116 and SW1116 cells transfected with inhibitor NC or miR-432-3p inhibitor. **(F)** Relative expression of miR-432-3p in HCT116 and SW1116 cells with stable shRNA-NC or sh-AC010789.1. **(G)** The schematic model showed the putative binding sites of miR-432-3p on AC010789.1. **(H)** Luciferase activity of AC010789.1-WT and AC010789.1-MUT reporter plasmid in HCT116 cells co-transfected with mimic NC or miR-432-3p mimic. **(I,J)** Expression levels of E-cadherin and vimentin were determined by western blot after transfection with mimic NC or miR-432-3p mimic in HCT116 and SW1116 cells. **(K,L)** Expression levels of E-cadherin and vimentin were determined by western blot after transfection with inhibitor NC or miR-432-3p inhibitor in HCT116 and SW1116 cells. **(M,N)** Transwell assay for investigating migration and invasion change after transfection with mimic NC or miR-432-3p mimic in HCT116 and SW1116 cells. **(O,P)** Transwell assay for investigating migration and invasion change after transfection with inhibitor NC or miR-432-3p inhibitor in HCT116 and SW1116 cells. Data are representative of three independent experiments and are presented as mean ± SEM. *P*-values were determined by two-tailed student’s *t*-test (*P < 0.05).

It has been reported that lncRNAs can act as miRNAs sponge to regulate downstream targets. Given that AC010789.1 is slightly enriched in the cytoplasm (Figure 4A), we explored whether AC010789.1 might also function via the ceRNA mechanism. Therefore, the putative candidate miRNAs binding AC010789.1 were predicted using LncBase Predicted v.2. Then, combined with the bioinformatic analysis results of Wang X. et al. (2018a), three miRNAs (miR-432-3p, miR-7854-3p, and miR-4796-3p) were identified to combine with AC010789.1. Among these miRNAs, miR-432-3p was most highly expressed after AC010789.1 silencing in HCT116 cells (Supplementary Figure 3A). Besides, the expression level of miR-432-3p negatively correlated with AC010789.1 expression level in 24 CRC tissues, while miR-4796-3p and miR-7854-3p were not correlated with AC010789.1 expression (Supplementary Figures 3B–D). Hence, we considered miR-432-3p as our potential candidate. In order to check the relationship between AC010789.1 and miR-432-3p, we used miR-432-3p mimic and inhibitor, whose mimicking or inhibitory activity was checked in HCT116 and SW1116 cells (Figures 4B,C). Transfection of miR-432-3p mimic into HCT116 and SW1116 cells decreased the expression of AC010789.1 (Figure 4D), and the opposite results were observed when miR-432-3p was downregulated (Figure 4E). Additionally, AC010789.1 knockdown led to significant upregulation of miR-432-3p expression (Figure 4F). Thus, to obtain direct evidence for the interaction between AC010789.1 and miR-432-3p, we subcloned wild-type (AC010789.1-WT) and mutated (AC010789.1-MUT) miR-432-3p binding sites into dual-luciferase reporters (Figure 4G). Dual-luciferase assays showed that miR-432-3p mimic reduced luciferase activities with the AC010789.1-WT expression vector, but not the AC010789.1-MUT (Figure 4H), which suggest that miR-432-3p is a direct target of AC010789.1.

We further analyzed the role of miR-432-3p in EMT, migration, and invasion of CRC cells. Results showed that overexpression of miR-432-3p enhanced the expression of E-cadherin and reduced the expression of vimentin in both protein and mRNA levels, whereas inhibition of miR-432-3p had the opposite effect (Figures 4I–L and Supplementary Figures 3E,F). As expected, the transwell assays confirmed that miR-432-3p mimic induced decreased migration and invasion capabilities (Figures 4M,N), whereas miR-432-3p inhibitor induced increased migration and invasion capabilities (Figures 4O,P), indicating that miR-432-3p was involved in EMT, migration, and invasion of CRC cells.

### MiR-432-3p Inhibits Migration and Invasion of CRC Cells Through Targeting ZEB1

**FIGURE 5:**
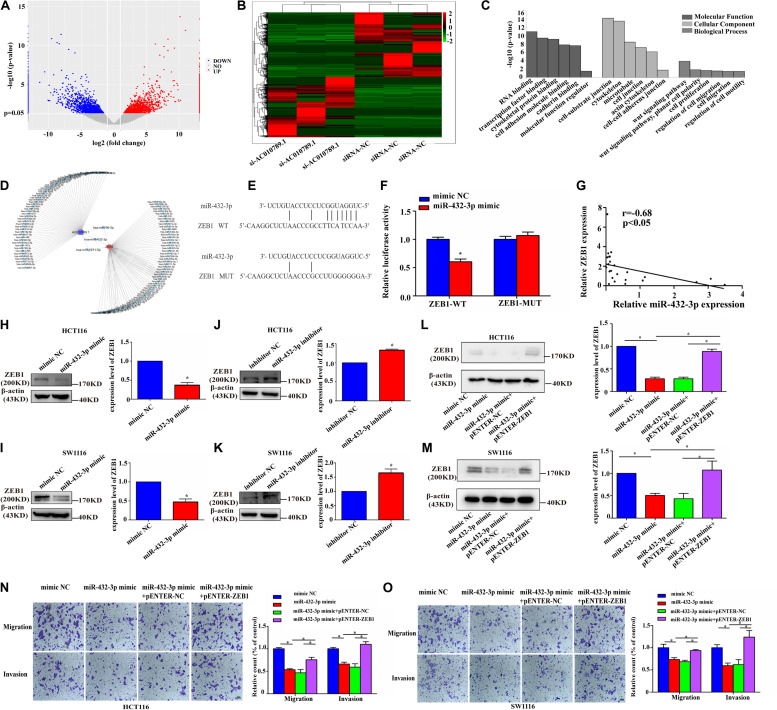
ZEB1 is a direct target of miR-432-3p in CRC migration and invasion. **(A,B)** RNA sequencing analysis of mRNAs from siRNA-NC and si-AC010789.1 cells were presented in a volcano plot and heatmap. **(C)** The Gene Ontology (GO) analysis and significantly enriched GO terms of changed targeted mRNAs in siRNA-NC and si-AC010789.1 cells on their molecular function, cellular component, and biological process. The statistically significant results were defined with –log10 (*P*-value) >1.40 as the cut-off criterion. **(D)** Schematic representation of the predicted binding miRNA for AC010789.1 and ZEB1. **(E)** The schematic model showed the putative binding sites of miR-432-3p on ZEB1. **(F)** ZEB1-WT and ZEB1-MUT reporter plasmid in HCT116 cells co-transfected with mimic NC or miR-432-3p mimic. **(G)** Correlation analysis of ZEB1 expression and miR-432-3p expression levels in 24 CRC tissues. **(H,I)** The protein level of ZEB1 in HCT116 and SW1116 cells transfected with mimic NC or miR-432-3p mimic. **(J,K)** The protein level of ZEB1 in HCT116 and SW1116 cells transfected with inhibitor NC or miR-432-3p inhibitor. **(L,M)** The protein level of ZEB1 in miR-432-3p overexpressed-HCT116 cells with or without ZEB1 upregulation. **(N,O)** Transwell assay for investigating migration and invasion change after transfection with mimic NC, miR-432-3p mimic, miR-432-3p mimic + pENTER-NC, miR-432-3p mimic + pENTER-ZEB1 in HCT116 and SW1116 cells, Scale bar: 100 μm. Data are representative of three independent experiments and are presented as mean ± SEM. *P*-values were determined by two-tailed student’s *t*-test or one-way ANOVA (*P < 0.05).

As stated earlier, miRNAs exert their multiple biological functions mainly by suppressing mRNA translation or degrading mRNA. After verifying that AC010789.1 was knocked down (Supplementary Figure 4A), we further examined the differential expression of mRNAs using RNA sequencing between si-AC010789.1 and siRNA-NC cells (Figures 5A,B), the differentially expressed mRNAs are presented in Supplementary Table S6. Gene Ontology (GO) enrichment analysis demonstrated that these candidate genes were mainly enriched in cell-substrate junction and cell migration (Figure 5C). Thus, we focused on EMT-related genes, such as snail1, snail2, vimentin, and ZEB1. However, a search of the TargetScan database indicated that only ZEB1-3′ UTR contained one region complementary to the “seed” region of miR-432-3p (Figures 5D,E). In the TCGA database, increased ZEB1 in CRC tissues was significantly correlated with higher lymph node metastasis and TNM stage (Supplementary Figures 4B,C). Dual-luciferase assays showed a significant decrease in luciferase activities following the co-transfection of miR-432-3p mimic and the ZEB1-WT, but not ZEB1-MUT (Figure 5F). Furthermore, the expression level of ZEB1 negatively correlated with the expression level of miR-432-3p in 24 CRC tissues (Figure 5G). Moreover, it was clear that miRNA mimic downregulated ZEB1 protein expression level, whereas the use of miRNA inhibitor upregulated ZEB1 protein expression level (Figures 5H–K); Besides, the ZEB1 mRNA expression level was decreased in the presence of miR-432-3p mimic, while miR-432-3p inhibitor could increase the expression level of ZEB1 mRNA (Supplementary Figures 4D,E). Additionally, the negative effect of miR-432-3p mimic on ZEB1 protein expression could be partially offset by the introduction of exogenous ZEB1 in HCT116 and SW1116 cells (Figures 5L,M); the qRT-PCR analysis showed that the downregulation of ZEB1 mRNA expression induced by miR-432-3p mimic could also be partially offset by the introduction of exogenous ZEB1 (Supplementary Figures 4F,G). Functionally, the introduction of ZEB1 could partially abrogate the inhibitory effect of miR-432-3p mimic on CRC cell migration and invasion capabilities (Figures 5N,O). These data supported the observation that ZEB1 was a downstream target of miR-432-3p.

### AC010789.1 Silencing Reduces CRC Migration, Invasion and EMT Through miR-432-3p-Dependent ZEB1 Downregulation

**FIGURE 6:**
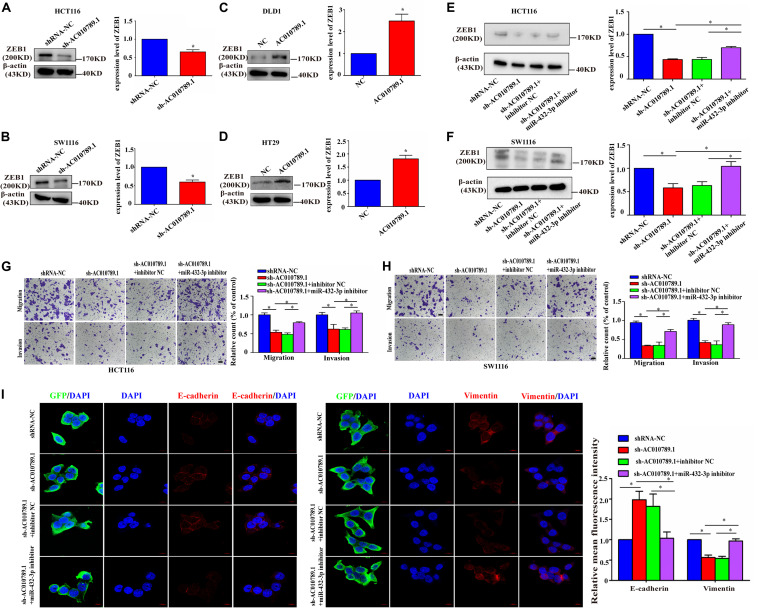
AC010789.1 promotes migration, invasion and EMT through miR-432-3p/ZEB1 axis. **(A,B)** The protein level of ZEB1 in HCT116 and SW1116 cells with AC010789.1 stable knockdown. **(C,D)** The protein level of ZEB1 in DLD1 and HT29 cells with AC010789.1 overexpression. **(E,F)** Expression level of ZEB1 was determined by western blot after transfection with shRNA-NC, sh-AC010789.1, sh-AC010789.1 + inhibitor NC, sh-AC010789.1 + miR-432-3p inhibitor in HCT116 and SW1116 cell. **(G,H)** Transwell assay for investigating migration and invasion change after transfection with shRNA-NC, sh-AC010789.1, sh-AC010789.1 + inhibitor NC, sh-AC010789.1 + miR-432-3p inhibitor in HCT116 and SW1116 cells, Scale bar: 100 μm. **(I)** The expression of E-cadherin (red), vimentin (red), and GFP (green) in HCT116 cells treated with shRNA-NC, sh-AC010789.1, sh-AC010789.1 + inhibitor NC, sh-AC010789.1 + miR-432-3p inhibitor were detected by immunofluorescence. Cells nuclei were stained with DAPI (blue). Error bars:10 μm. Data are representative of three independent experiments and are presented as mean ± SEM. *P*-values were determined by two-tailed student’s *t*-test or one-way ANOVA (*P < 0.05).

Then, we further tested whether AC010789.1 is involved in migration, invasion, and EMT through the miR-432-3p/ZEB1 axis. Initially, AC010789.1 knockdown led to the significant downregulation of ZEB1 expression, whereas its overexpression resulted in the upregulation of ZEB1 (Figures 6A–D). We also observed that the ZEB1 mRNA expression was increased in the presence of AC010789.1 overexpression, while AC010789.1 silencing could decrease the expression level of ZEB1 mRNA as observed after performing qRT-PCR based experiments (Supplementary Figure 5A). Besides, the expression level of ZEB1 positively correlated with the expression level of AC010789.1 in 24 CRC tissues (Supplementary Figure 5B). These results confirmed our hypothesis that AC010789.1 did positively regulate the ZEB1 expression level. Furthermore, downregulation of ZEB1 protein expression induced by AC010789.1 silencing was partly abrogated by the co-transfection of miR-432-3p inhibitor in HCT116 and SW1116 cells (Figures 6E,F). Moreover, the qRT-PCR analysis showed that the downregulation of ZEB1 mRNA expression induced by AC010789.1 silencing could also be partially offset by the inhibition of miR-432-3p (Supplementary Figures 5C,D). Functionally, we observed that the weakened migration and invasion capabilities of AC010789.1 knockdown were partially reversed by co-transfection with miR-432-3p inhibitor in HCT116 and SW1116 cells (Figures 6G,H). Besides, the immunofluorescence analysis indicated that the upregulation of E-cadherin and the downregulation of vimentin induced by AC010789.1 silencing could be partly rescued after miR-432-3p downregulation in GFP positive cells (Figure 6I). From these collective observations, we surmised that AC010789.1 served as a sponge for miR-432-3p to regulate ZEB1 expression and promote cell metastasis and EMT via the ceRNA mechanism in CRC cell lines.

### Wnt/β-Catenin Signaling Pathway Contributes to AC010789.1-Mediated Migration and Invasion

**FIGURE 7:**
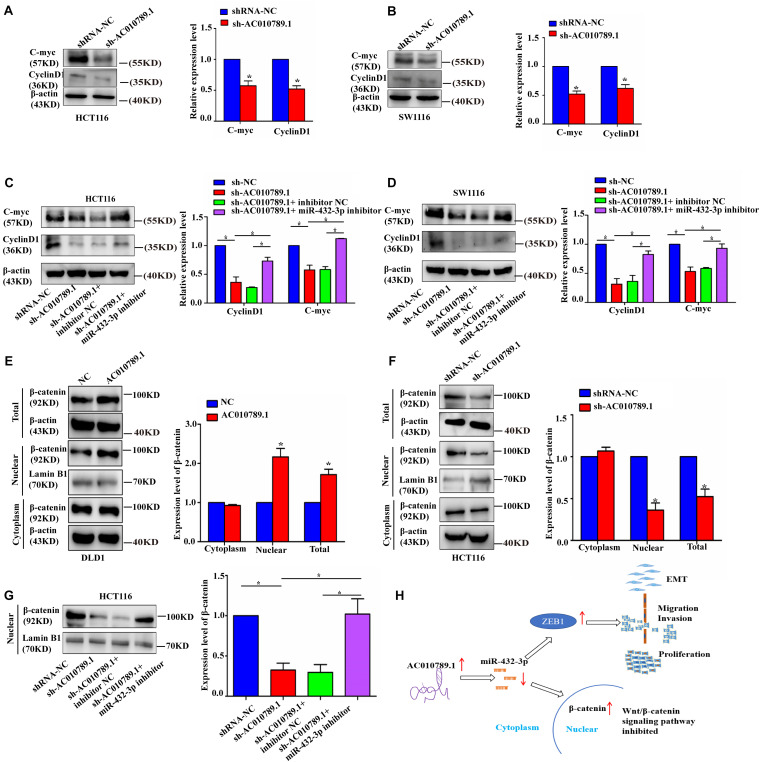
AC010789.1 expression level correlates with Wnt/β-catenin pathway activation in CRC cells. **(A, B)** The protein expression levels of Wnt-responsive genes in HCT116 and SW1116 cells with AC010789.1 stable knockdown. **(C, D)** Western blot analysis of C-myc and cyclin D1 in HCT116 and SW1116 cells transfected with shRNA-NC, sh-AC010789.1, sh-AC010789.1 + inhibitor NC, sh-AC010789.1 + miR-432-3p inhibitor. **(E)** Western blot analysis showed the β-catenin protein levels of total, nuclear, and cytoplasmic in DLD1 cells with AC010789.1 overexpression. **(F)** Western blot analysis showed the β-catenin protein levels of total, nuclear, and cytoplasmic in HCT116 cells with AC010789.1 knockdown. **(G)** Western blot analysis showed the β-catenin protein level of nucleus in HCT116 cells transfected with shRNA-NC, sh-AC010789.1, sh-AC010789.1 + inhibitor NC, sh-AC010789.1 + miR-432-3p inhibitor. **(H)** Schematic diagram of the regulatory mechanism of AC010789.1 on CRC proliferation, metastasis and EMT. AC010789.1 is upregulated in CRC, and results in downregulation of miR-432-3p. The decreased expression of miR-432-3p leads to an upregulation of ZEB1 whose function is to promote EMT, migration and proliferation. The decreased expression of miR-432-3p also results in increased levels of nuclear β-catenin, resulting in increased Wnt/β-catenin signaling. Data are representative of three independent experiments and are presented as mean ± SEM. *P*-values were determined by two-tailed student’s *t*-test or one-way ANOVA (*P < 0.05).

According to the results of GO biological process enrichment analysis, AC010789.1 was associated with the Wnt/β-catenin pathway in CRC. Then, we measured the expression levels of Wnt/β-catenin pathway target genes to evaluate the activation of the Wnt/β-catenin pathway in AC010789.1-silenced cells. The results showed that mRNA expression levels of TCF1, JUN, CD44, C-myc, and cyclin D1 were decreased with AC010789.1 knockdown (Supplementary Figures 6A,B). Consistent with these results, the protein expression levels of C-myc and cyclin D1 were also decreased (Figures 7A,B). In addition, the inhibitory role of sh-AC010789.1 on C-myc and cyclin D1 protein expression could be partially restored by co-transfection with miR-432-3p inhibitor (Figures 7C,D). Considering that activated β-catenin could be translocated to the nucleus and control the transcription of target genes, we then analyzed the change of β-catenin in the nucleus after AC010789.1 knockdown. In order to verify the purity of the nuclear protein, we tested the expression of E-catenin in the nucleus and the expression of lamin B1 in the cytoplasm. Results showed that there was no expression of E-cadherin in the nuclear protein and no expression of lamin B1 in the cytoplasm, showing that the nuclear and cytoplasmic proteins did not contaminate each other (Supplementary Figures 6C,D). Furthermore, Western blot analysis of β-catenin showed that upregulated AC010789.1 expression resulted in an increase in the expression levels of total and nuclear β-catenin expression, while silencing AC010789.1 had the opposite effect. However, there was no significant difference in the cytoplasmic protein level of β-catenin (Figures 7E,F). In addition, the inhibitory role of sh-AC010789.1 on nuclear β-catenin expression could be partially restored by co-transfection with miR-432-3p inhibitor (Figure 7G). These data suggested that AC010789.1 could regulate the Wnt/β-catenin pathway activation partly through miR-432-3p.

## Discussion

In recent years, the functions of lncRNAs have drawn more and more attention. A significant number of studies have indicated that lncRNAs play essential roles in all steps of carcinogenesis and tumor progression, and lncRNAs could be used as new prognostic markers (Li et al., 2018; Wang Z.Y. et al., 2018). MALAT1 was reported to suppress breast cancer metastasis in the transgenic, xenograft, and syngeneic models (Kim et al., 2018). Zhou and colleagues reported that lncRNA GMAN was increased in gastric tumors and associated with survival (Zhuo et al., 2019). In this study, we characterized that AC010789.1 was expressed at a low level in healthy tissues but a high level in CRC tissues. Also, the high expression of AC010789.1 was indicative of poor prognosis and associated with lymph node metastasis, which was consistent with previous studies (Wang X. et al., 2018).

To date, accumulating evidence has indicated that lncRNAs can function as ceRNAs for miRNAs in cancer (Salmena et al., 2011). For example, lncRNA UICLM promoted CRC liver metastasis by acting as a ceRNA for miR-215 to regulate ZEB2 expression (Chen et al., 2017). Qu et al. (2016) identified that lncARSR served as a ceRNA for miR-34 and miR-449 to regulate AXL and c-MET expression in renal cell cancer cells, and thus reinforce the sunitinib resistance. Likewise, in high glucose-induced vascular endothelial cells, lncRNA CA7-4 promoted autophagy and apoptosis through binging miR-877-3P and miR-5680 (Zhao et al., 2019). Emerging evidence is pointing to the significance of a large number of ncRNA carrying miRNA target sites—so-called miRNA “sponges”—for sequestering different miRNA molecules, thus de-repressing other target transcripts (Bak and Mikkelsen, 2014; Liao et al., 2020). Herein, we identified that AC010789.1 acted as a molecular sponge for miR-432-3p. Many reports have been reported that miR-432-3p played an essential role in the post-transcriptional regulation of several diseases (Huang et al., 2012; Ma et al., 2017). However, the roles of miR-432-3p in CRC remain unclear. In the current study, we found that miR-432-3p could inhibit CRC cell migration and invasion. Also, increased expression of miR-432-3p significantly reduced the expression of vimentin, and conversely enhanced the expression of E-cadherin. More importantly, the rescue experiment showed that miR-432-3p inhibitor significantly attenuated the effects of sh-AC010789.1 on cell migration, invasion, and EMT, indicating that miR-432-3p was involved in the effect of AC010789.1 in regulating CRC cells functions.

Emerging studies have documented that the 5′ region of a miRNA (known as the “seed” region) has particular importance in targeting. It is the region that is most frequently complementary to target sites in 3′ untranslated region (3′-UTRs) and is sufficient to suppressing mRNA translation or degrading mRNA (Lewis et al., 2005; Fabian et al., 2010; Hassan et al., 2013). Based on this, we used RNA sequencing to explore downstream targets of miR-432-3p, which is essential for AC010789.1-mediated oncogenic function. When analyzing the results of RNA sequencing, we found that there are substantial differences within the group. This may be due to the different generations of cells used in each biological repeat. By excluding the genes with significant differences within the group, we identified a close association between AC010789.1 and ZEB1, a vital gene involved in cell migration, invasion, and EMT. Numerous tumor-profiling studies point to the miR-200-ZEB1 axis as crucial in regulating tumor progression and metastasis (Hill et al., 2013; Title et al., 2018). As it is well known that a single mRNA can be bound by multiple miRNAs (Ebert and Sharp, 2010), in the current study, we verified that ectopic expression of miR-432-3p could reduce ZEB1 expression in CRC cells. In contrast, inhibition of miR-432-3p could enhance ZEB1 expression. As expected, the exogenous introduction of ZEB1 could partially attenuate the effects of miR-432-3p mimic on migration, invasion, and EMT, and downregulation of miR-432-3p significantly reversed the effects of silencing AC010789.1 on migration, invasion, EMT and ZEB1 expression. All of the above results suggested that ZEB1 was a direct target of miR-432-3p. These findings enhance our understanding of the AC010789.1-miR-432-3p-ZEB1 axis during CRC progression.

The Wnt/β-catenin pathway is evolutionarily well-conserved, and aberrant activation of this pathway can cause abnormal cell growth and malignant transformation (Clevers and Nusse, 2012; Sun et al., 2020). Wang et al. (2019) revealed that the novel lncRNA OTUD6B-AS1 decreased the activity of the Wnt/β-catenin pathway and suppressed the expression of EMT-related proteins. In line with this result, LINC01133 attenuated the EMT and metastatic abilities of gastric cancer cells by inactivating the APC/Wnt/β-catenin pathway (Yang et al., 2018). Herein, our results showed that sh-AC010789.1 substantially decreased the Wnt-responsive genes and β-catenin nuclear signals in HCT116 and SW1116 cells, and the trend of action in the two cell lines were the same. Because of the difference in the biological characteristics of the two cell lines, their degrees of response to AC010789.1 knockdown were significantly different. Further analysis showed that AC010789.1 knockdown-induced downregulation of total and nuclear β-catenin expression, and the opposite result was observed when AC010789.1 was upregulated. However, there was no significant difference in the cytoplasmic protein level of β-catenin; the detailed molecular mechanism is not currently understood in depth. Besides, AC010789.1 knockdown-induced downregulation of nuclear β-catenin expression could be partially reversed by co-transfection with miR-432-3p inhibitor, but the exact mechanism by which AC010789.1 acts on the Wnt/β-catenin signaling pathway requires further study. In sum, our results suggested that AC010789.1 could regulate the Wnt/β-catenin pathway activation through miR-432-3p.

In summary, our data identified that AC010789.1 silencing reduced CRC progression through miRNA-432-3p-dependent ZEB1 downregulation and suppression of the Wnt/β-catenin signaling pathway (Figure 7H). Our findings provide a novel glimpse of the mechanism of AC010789.1 to promote the malignant phenotypes of CRC cells, which will shed light on the future development of lncRNA-based CRC therapies.

## Data Availability Statement

The raw and processed data of Next Generation Sequencing have been deposited into the Gene Expression Omnibus (GEO) database under accession number GSE158445.

## Ethics Statement

The studies involving human participants were reviewed and approved by the Committee for Ethical Review of Research involving Human Subjects of The Second Hospital, Cheeloo College of Medicine, Shandong University. The patients/participants provided their written informed consent to participate in this study. The animal study was reviewed and approved by Institutional Animal Care and Use Committee of The Second Hospital, Cheeloo College of Medicine, Shandong University. Written informed consent was obtained from the individual(s) for the publication of any potentially identifiable images or data included in this article.

## Author Contributions

WD, LD, and CW initiated, organized, and supervised the study. WD and XK performed most of the experiments, collected, and analyzed the data. PL, TL, and YZ provided technical support. YW, HB, and JL critically revised the manuscript. All authors have read and approved the final version of the manuscript.

## Conflict of Interest

The authors declare that the research was conducted in the absence of any commercial or financial relationships that could be construed as a potential conflict of interest.

## Footnotes

1 http://cancergenome.nih.gov/publications/publicationguidelines

2 https://tcga-data.nci.nih.gov/
