# Supplementary material for: LncRNA AC010789.1 Promotes Colorectal Cancer Progression by Targeting MicroRNA-432-3p/ZEB1 Axis and the Wnt/β-Catenin Signaling Pathway
Source: Front Cell Dev Biol. 2020 Oct 15;8:565355. doi: 10.3389/fcell.2020.565355 (PMC7593606; doi:10.3389/fcell.2020.565355)
Supplement: Supplementary file 1 [file Data_Sheet_1.docx]

Supplementary Material

# Supplementary Data

Supplementary Material should be uploaded separately on submission. Please include any supplementary data, figures and/or tables. All supplementary files are deposited to FigShare for permanent storage and receive a DOI.

Supplementary material is not typeset so please ensure that all information is clearly presented, the appropriate caption is included in the file and not in the manuscript, and that the style conforms to the rest of the article. To avoid discrepancies between the published article and the supplementary material, please do not add the title, author list, affiliations or correspondence in the supplementary files.

# Supplementary Figures and Tables

For more information on Supplementary Material and for details on the different file types accepted, please see [here](http://home.frontiersin.org/about/author-guidelines#SupplementaryMaterial). Figures, tables, and images will be published under a Creative Commons CC-BY licence and permission must be obtained for use of copyrighted material from other sources (including re-published/adapted/modified/partial figures and images from the internet). It is the responsibility of the authors to acquire the licenses, to follow any citation instructions requested by third-party rights holders, and cover any supplementary charges.

## Supplementary Figures


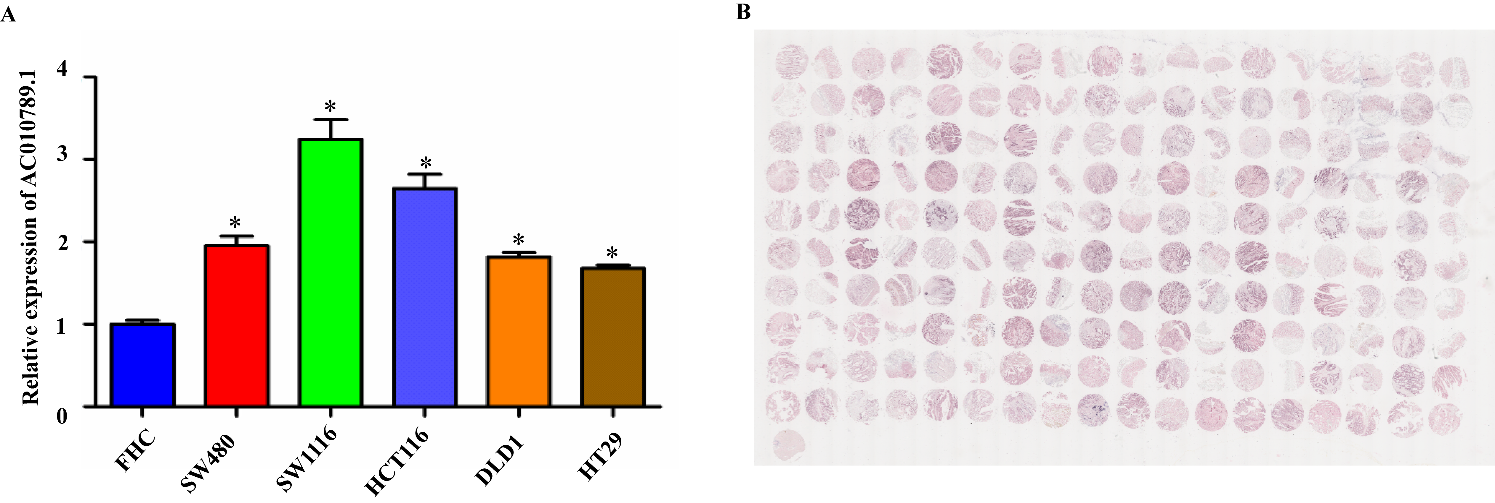


**Supplementary Figure S1. The expression level of AC010789.1 in CRC cell lines and tissues. (A)** The qRT-PCR analysis of AC010789.1 expression levels in CRC cell lines and FHC. Data are presented as mean ±SEM from triplicate independent experiments. *P*-values were determined by one-way ANOVA (* *p* < 0.05). **(B)** Overview of the tissue microarray.


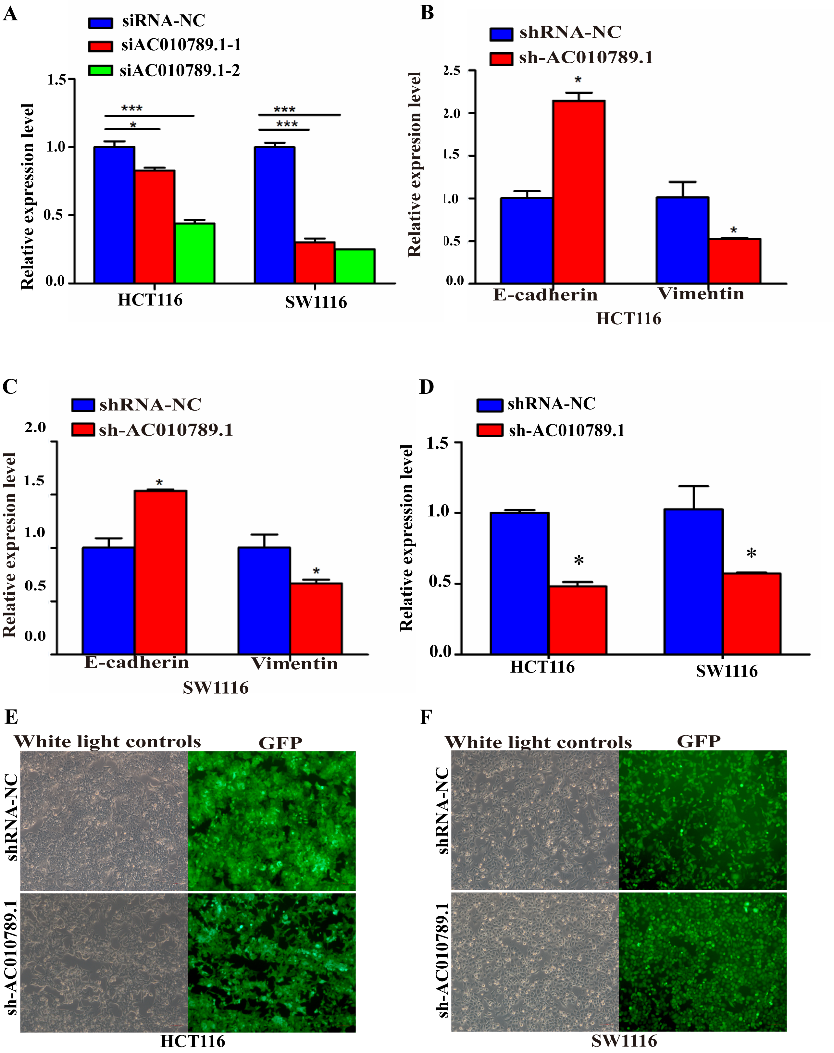


**Supplementary Figure S2. Silencing AC010789.1 inhibits EMT of CRC cells in vitro.** **(A)** The qRT-PCR was performed to verify the relative expression of AC010789.1 in HCT116 and SW1116 cells transfected with two independent siRNAs targeting AC010789.1. **(B, C)** The relative expression levels of E-cadherin and vimentin were determined by qRT-PCR in HCT116 and SW1116 cells with AC010789.1 knockdown. **(D)** Knockdown efficiency verification of sh-NC and sh-AC010789.1 stable cell lines. **(E, F)** The expression of GFP (green) was observed under a fluorescence microscope to determine the transfection efficiency in HCT116 and SW1116 cells. Scale bar: 100μm. Data are presented as mean ±SEM from triplicate independent experiments. *P*-values were determined by one-way ANOVAs or two-tailed Student’s t-test ( **p* < 0.05,***p* < 0.01, *** *p* < 0.001).


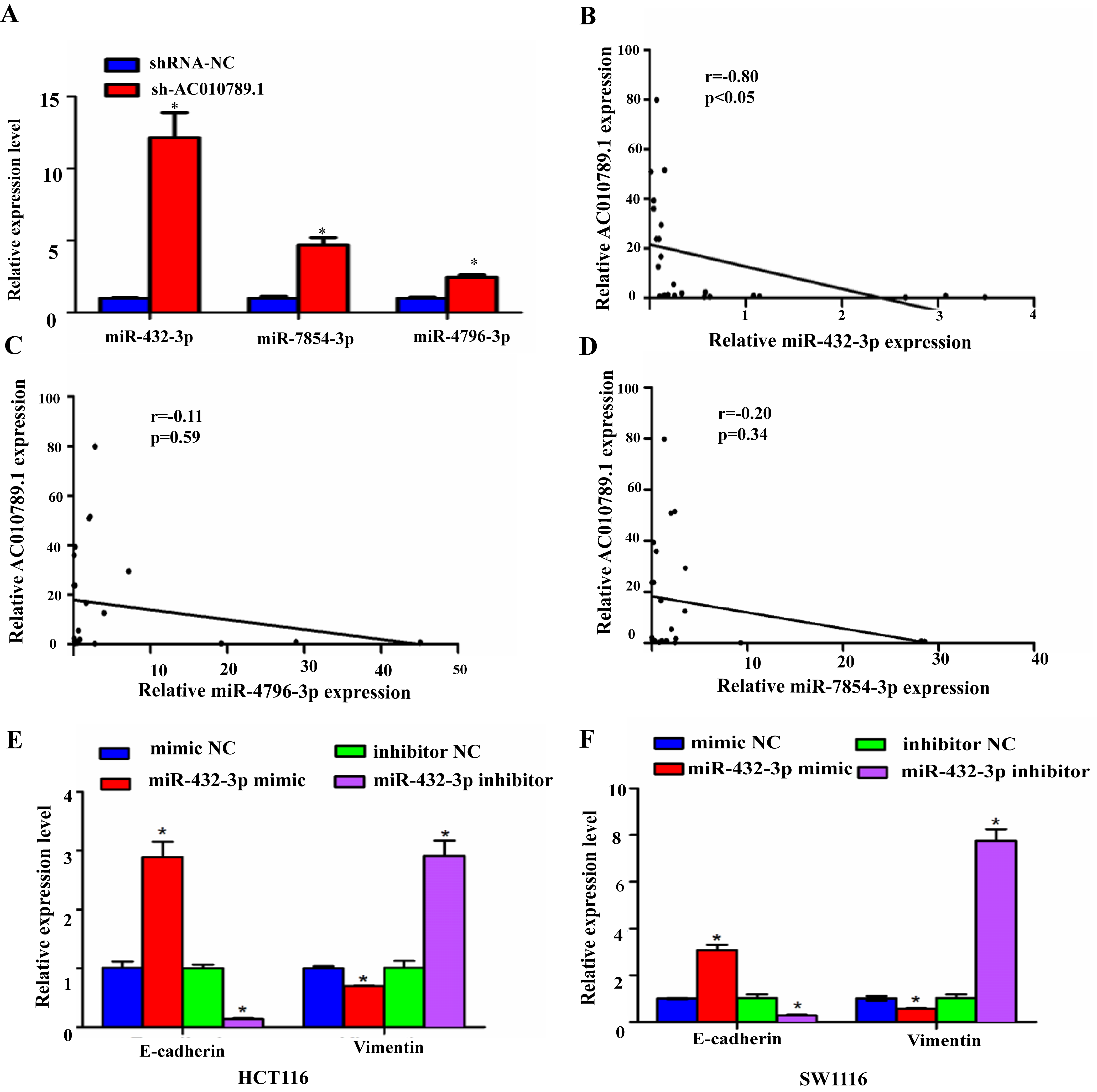


**Supplementary Figure S3. MiR-432-3p directly binds to AC010789.1 producing anti-EMT effect in CRC cells. (A)** The qRT-PCR was performed to verify the relative expression of miR-432-3p, miR-7854-3p and miR-4796-3p in HCT116 cells transfected with shRNA-NC and sh-AC010789.1. **(B)** The correlation between AC010789.1 expression and miR-432-3p expression levels were measured in CRC tissues by Spearman correlation analysis (n=24). **(C)** The correlation analysis of AC010789.1 expression and miR-4796-3p expression levels were measured in CRC tissues by Spearman correlation analysis (n=24). **(D)** The correlation analysis of AC010789.1 expression and miR-7854-3p expression levels were measured in CRC tissues by Spearman correlation analysis (n=24). **(E, F)** Expression levels of E-cadherin and vimentin were determined by qRT-PCR after transfecting with mimic NC, miR-432-3p mimic, inhibitor NC and miR-432-3p inhibitor in HCT116 and SW1116 cells. Data are presented as mean ±SEM from triplicate independent experiments. *P*-values were determined by two-tailed Student’s t-test (* *p* < 0.05).


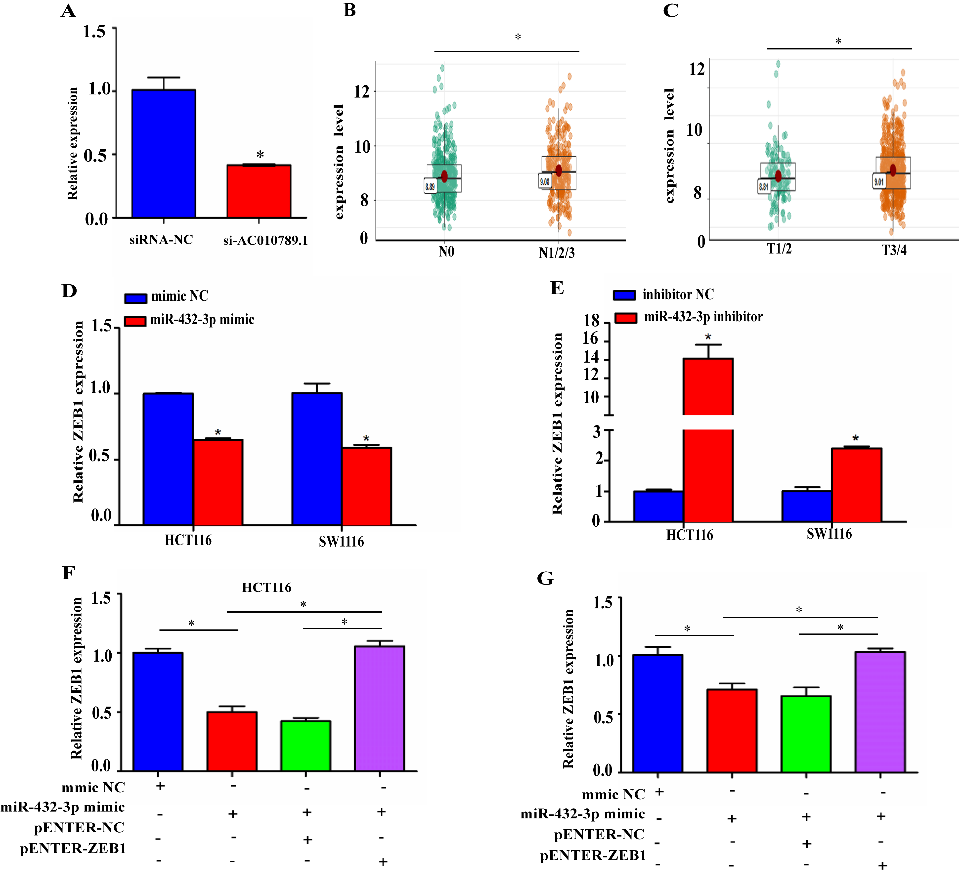


**Supplementary Figure S4.** **ZEB1 is a direct target of miR-432-3p in CRC migration and invasion. (A)** Knockdown efficiency verification of RNA-seq samples. **(B)** ZEB1 expression level in lymph node metastasis and non-lymph node metastasis of CRC tissues in the TCGA database. **(C)** ZEB1 expression level in T1/2 stage and T3/4 stage of CRC tissues in the TCGA database. **(D)** The mRNA level of ZEB1 in HCT116 and SW1116 cells transfected with mimic NC or miR-432-3p mimic. **(E)** The mRNA level of ZEB1 in HCT116 and SW1116 cells transfected with inhibitor NC or miR-432-3p inhibitor. **(F)** The mRNA level of ZEB1 in miR-432-3p overexpressed-HCT116 cells with or without ZEB1 upregulation. **(G)** The mRNA level of ZEB1 in miR-432-3p overexpressed-SW1116 cells with or without ZEB1 upregulation. Data are presented as mean ±SEM from triplicate independent experiments. *P*-values were determined by two-tailed Student’s t-test or one-way ANOVAs (* *p* < 0.05).


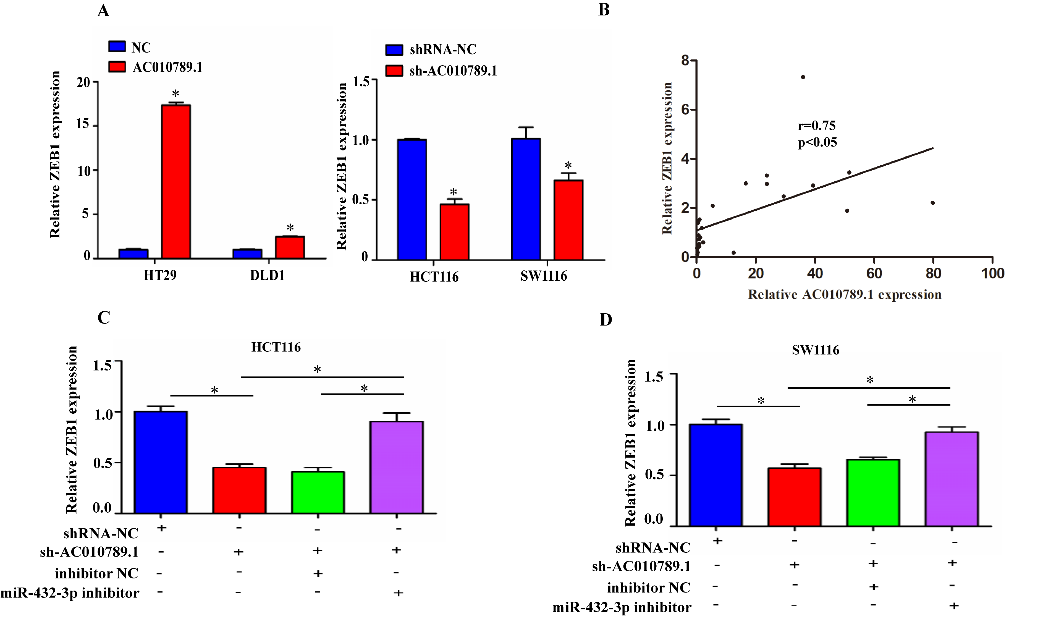


**Supplementary Figure S5. AC010789.1 positively regulates the expression of ZEB1. (A)**The mRNA level of ZEB1 in CRC cells with AC010789.1 overexpression or knockdown **(B)** Correlation analysis of AC010789.1 expression and ZEB1 expression levels in CRC tissues (n=24). **(C)** The mRNA level of ZEB1 in AC010789.1 knockdown-HCT116 cells with or without miR-432-3p inhibitor. **(D)** The mRNA level of ZEB1 in AC010789.1 knockdown-SW1116 cells with or without miR-432-3p inhibitor. Data are presented as mean ±SEM from triplicate independent experiments. *P*-values were determined by two-tailed Student’s t-test or one-way ANOVAs (* *p* < 0.05).


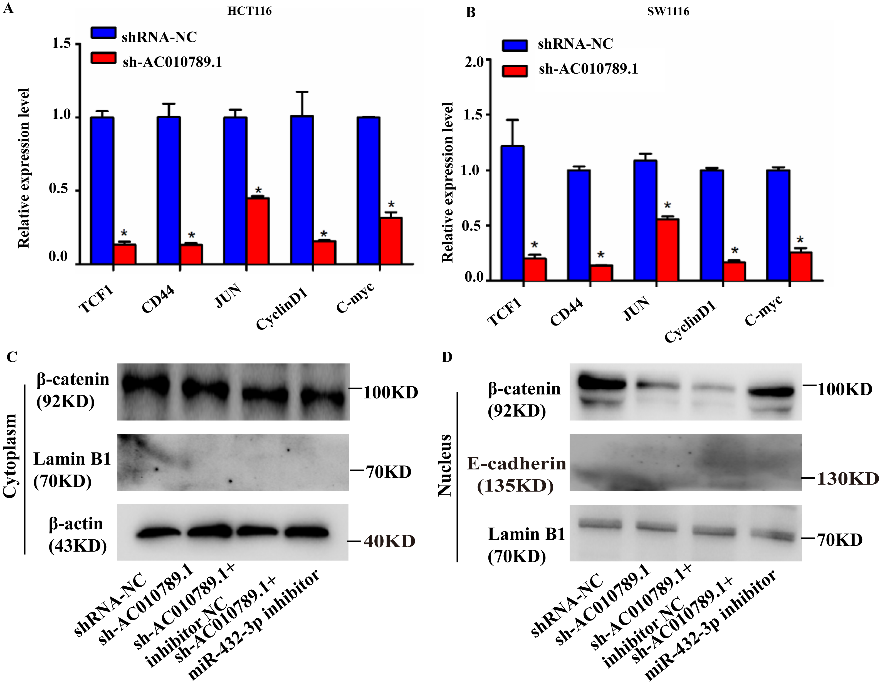


**Supplementary Figure S6. AC010789.1 expression level correlates with** **Wnt/β-catenin pathway activation in CRC cells. (A, B)** The mRNA expression levels of Wnt-responsive genes in HCT116 and SW1116 cells with AC010789.1 knockdown. **(C)** The expression levels of β-actin, β-catenin and Lamin B1 in the cytoplasm of HCT116 cells. **(D)** The expression levels of Lamin B1, β-catenin and E-cadherin in the nucleus. Data are presented as mean ±SEM from triplicate independent experiments. *P*-values were determined by two-tailed Student’s t-test (* *p* < 0.05).

## Supplementary Tables

Supplementary Table S1. Characteristics of 86 CRC patients

| **variables** | **No. of patients (%)** |
| --- | --- |
| **Age** |  |
| <63 | 41 (47.7) |
| ≥63 | 45 (52.3) |
| **Sex** |  |
| Male | 50 (58.1) |
| Female | 36 (41.9) |
| **Tumor stage** |  |
| T1/2 | 28 (32.6) |
| T3/4 | 58 (67.4) |
| **Lymph node metastasis** |  |
| Negative | 49 (57) |
| Positive | 37 (43) |

Supplementary Table S2. Clinicopathological characteristics of patients with CRC used for ISH assay

| **variables** |  | **No. of patients (%)** |
| --- | --- | --- |
|  |  |  |
| **Age (year)** |  |  |
|  | ≤65 | 45 (44.6) |
|  | ＞65 | 56 (55.4) |
| **T stage** |  |  |
|  | T1/T2 | 6 (5.9) |
|  | T3/T4 | 94 (93.1) |
|  | Not record | 1 (1) |
| **TNM stage** |  |  |
|  | Ι/II | 61(60.4) |
|  | III/IV | 40 (39.6) |
| **N stage** |  |  |
|  | N0 | 61 (60.4) |
|  | N1/N2 | 40 (39.6) |
| **M stage** |  |  |
|  | M0 | 100 (99) |
|  | M1 | 1 (1) |
| **Sex** |  |  |
|  | Female | 51 (50.5) |
|  | Male | 50 (49.5) |
| **grade** |  |  |
|  | I/ II | 84 (83.2) |
|  | III/Ⅳ | 17 (16.8) |

Supplementary Table S3. Primer sequences for qRT-PCR.

| **Gene** | **Forward (5’-3’)** | **Reverse (5’-3’))** |
| --- | --- | --- |
| β-Actin | CATGTACGTTGCTATCCAGGC | CTCCTTAATGTCACGCACGAT |
| AC010789.1 | TGCATCCCTGGCAATACTCAG | GGAGTGCTGTGCATTCATTGG |
| ZEB1 | GCACAACCAAGTGCAGAAGA | GCCTGGTTCAGGAGAAGATG |
| E-cadherin | GGGGTCTGTCATGGAAGGTG | GAAACTCTCTCGGTCCAGCC |
| Vimentin | ACCGCACACAGCAAGGCGAT | CGATTGAGGGCTCCTAGCGGTT |
| β-catenin | GGCTTGGAATGAGACTGCTG | GGTCCATACCCAAGGCATCC |
| Cyclin D1 | GATGCCAACCTCCTCAACGA | GGAAGCGGTCCAGGTAGTTC |
| c-MYC | CCCTCCACTCGGAAGGACTA | GCTGGTGCATTTTCGGTTGT |
| JUN | AGTGCCGAAAAAGGAAGCTG | CTGCTGCGTTAGCATGAGTT |
| TCF1 | AGGTGCGTGTCTACAACTGG | TGGACCTTACTGGGGGAGAG |
| CD44 | CCCAGCAACCCTACTGATGA | TGATCCAGGGACTGTCTTCG |
| U6 | CTCGCTTCGGCAGCACA | AACGCTTCACGAATTTGCGT |

**Supplementary Table S4. Clinical variables for colorectal cancer in TCGA database.**

| **variables** | **Total (647)** |
| --- | --- |
| **Age (years)** |  |
| ≥65 | 388 |
| ＜65 | 256 |
| Not report | 3 |
| **Gender** |  |
| Male | 342 |
| Female | 302 |
| not report | 3 |
| **TNM stage** |  |
| I+II | 353 |
| III+IV | 275 |
| Not report | 19 |
| **Lymph node metastasis** |  |
| Negative | 369 |
| Positive | 271 |
| Not report | 7 |

Supplementary Table S5. Correlation between tissue lncRNA concentrations and clinicopathological characteristics of patients with CRC [median (interquartile range)]

| **Parameters** | **Total cases** | **AC010789.1** | **P** |
| --- | --- | --- | --- |
| **Age** | | | 0.60 |
| <63 | 41 | 5.35(0.98-16.1) |  |
| ≥63 | 45 | 5.50(0.86-40.1) |  |
| **Sex** | | | 0.91 |
| Male | 50 | 5.42(1.00-21.97) |  |
| Female | 36 | 5.30(0.60-29.21) |  |
| **Tumor stage** | | | 0.97 |
| T1-T2 | 28 | 4.57(0.89-34.32) |  |
| T3-T4 | 58 | 5.68(1.00-19.50) |  |
| **Lymph node metastasis** | | | 0.02* |
| Negative | 49 | 2.23(0.72-16.38) |  |
| Positive | 37 | 11.95(3.14-31.64) |  |
