# Supplementary figures and images for: LncRNA AC010789.1 Promotes Colorectal Cancer Progression by Targeting MicroRNA-432-3p/ZEB1 Axis and the Wnt/β-Catenin Signaling Pathway
Source: Front Cell Dev Biol. 2020 Oct 15;8:565355. doi: 10.3389/fcell.2020.565355 (PMC7593606; doi:10.3389/fcell.2020.565355)

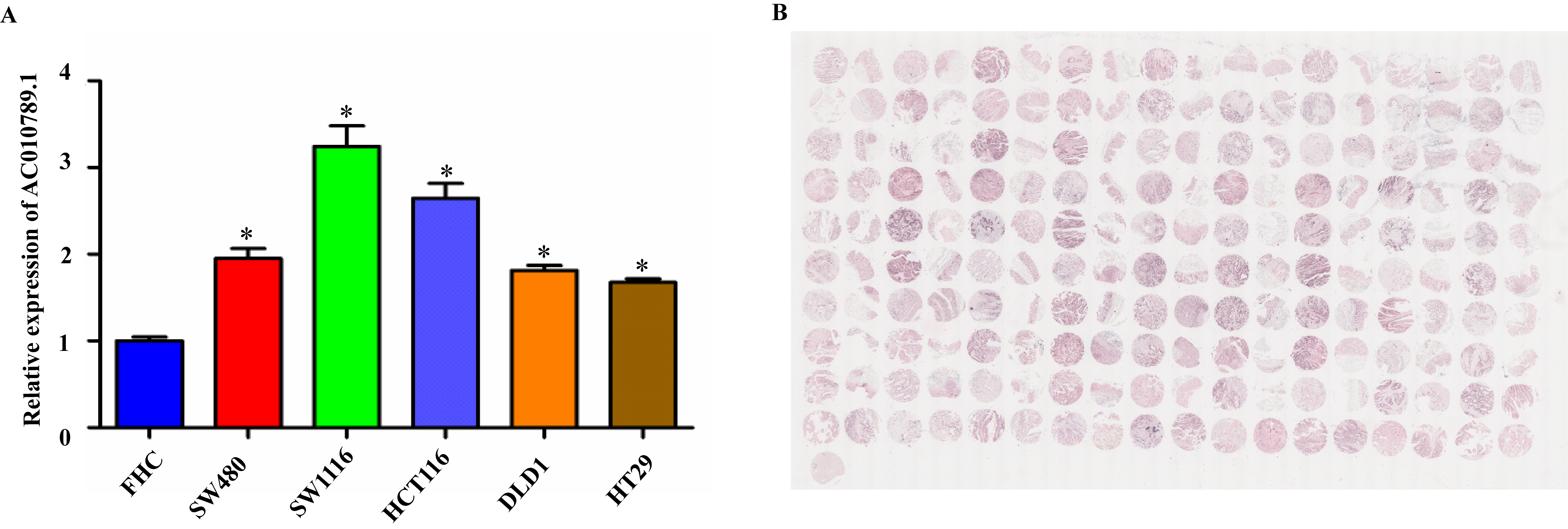

Supplement: Supplementary file 2 [file Image_1.TIF]

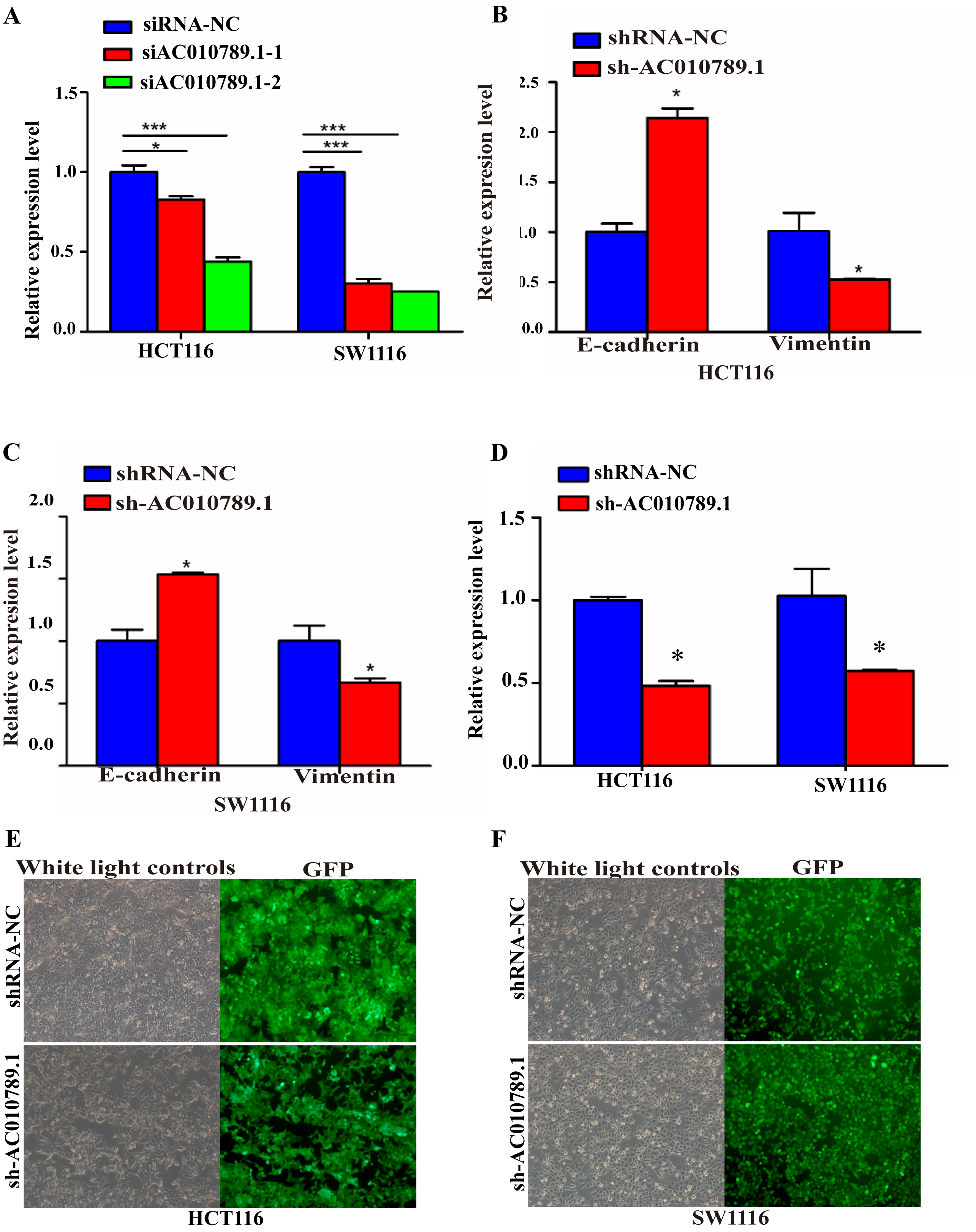

Supplement: Supplementary file 3 [file Image_2.TIF]

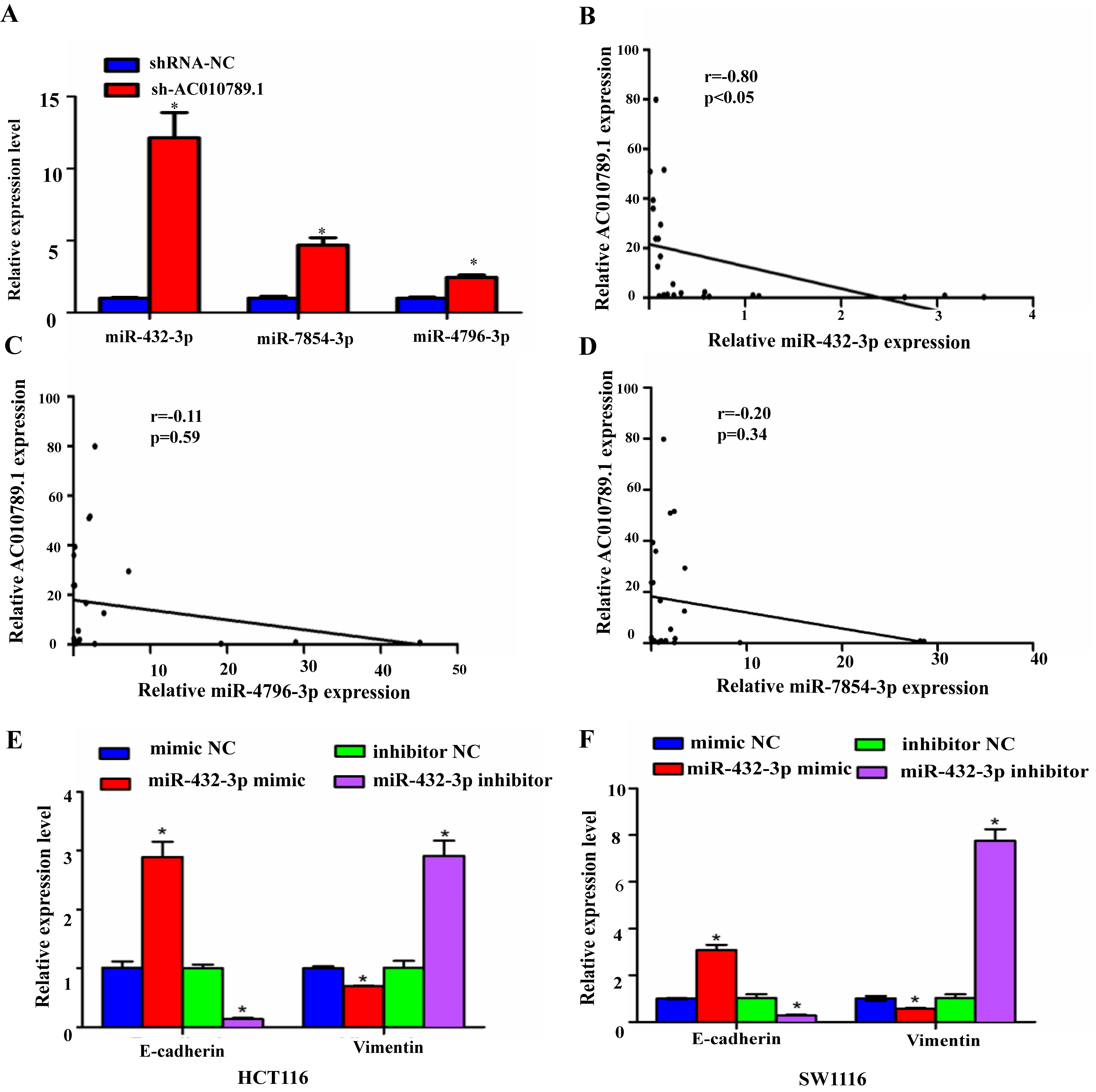

Supplement: Supplementary file 4 [file Image_3.TIF]

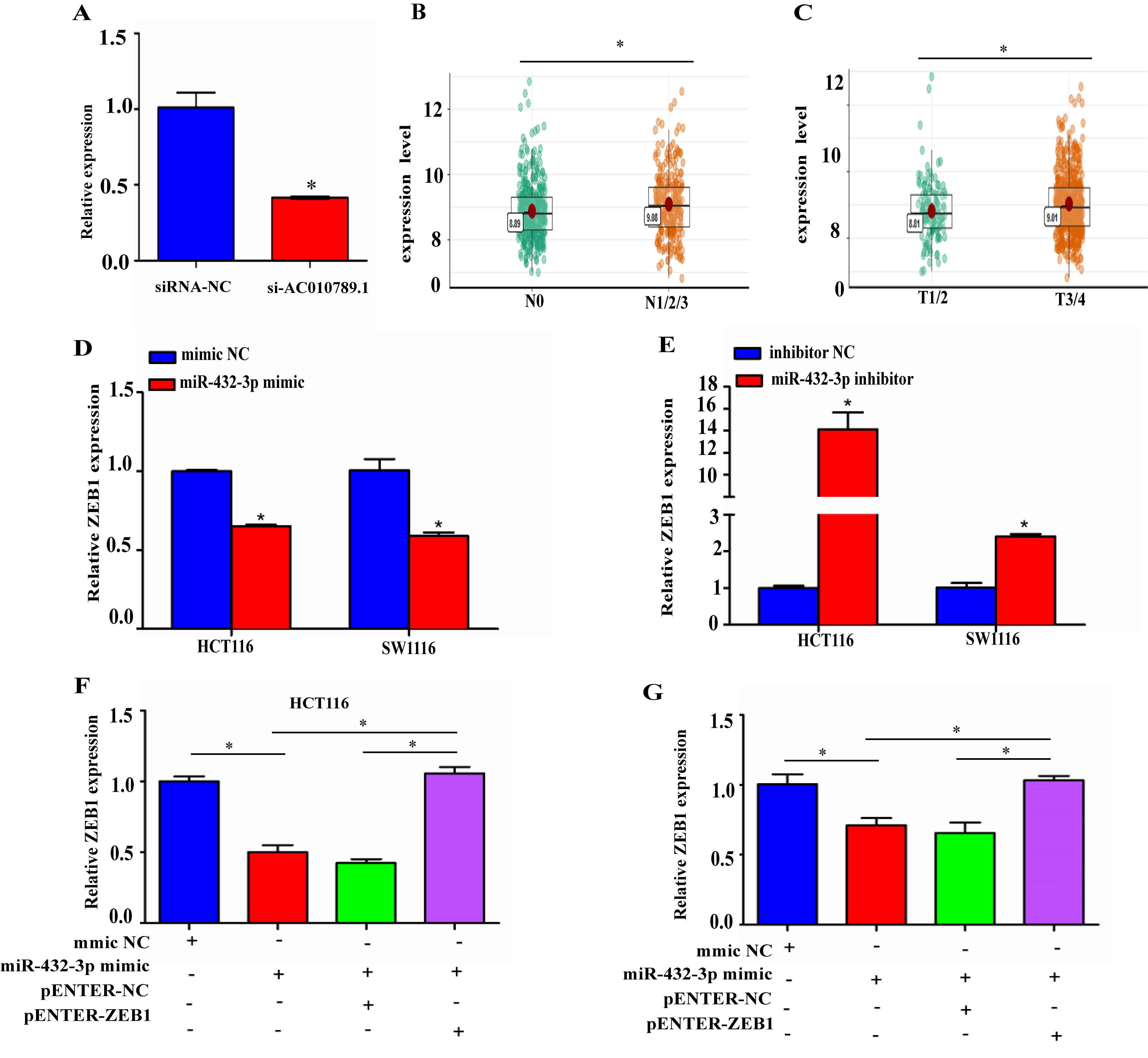

Supplement: Supplementary file 5 [file Image_4.TIF]

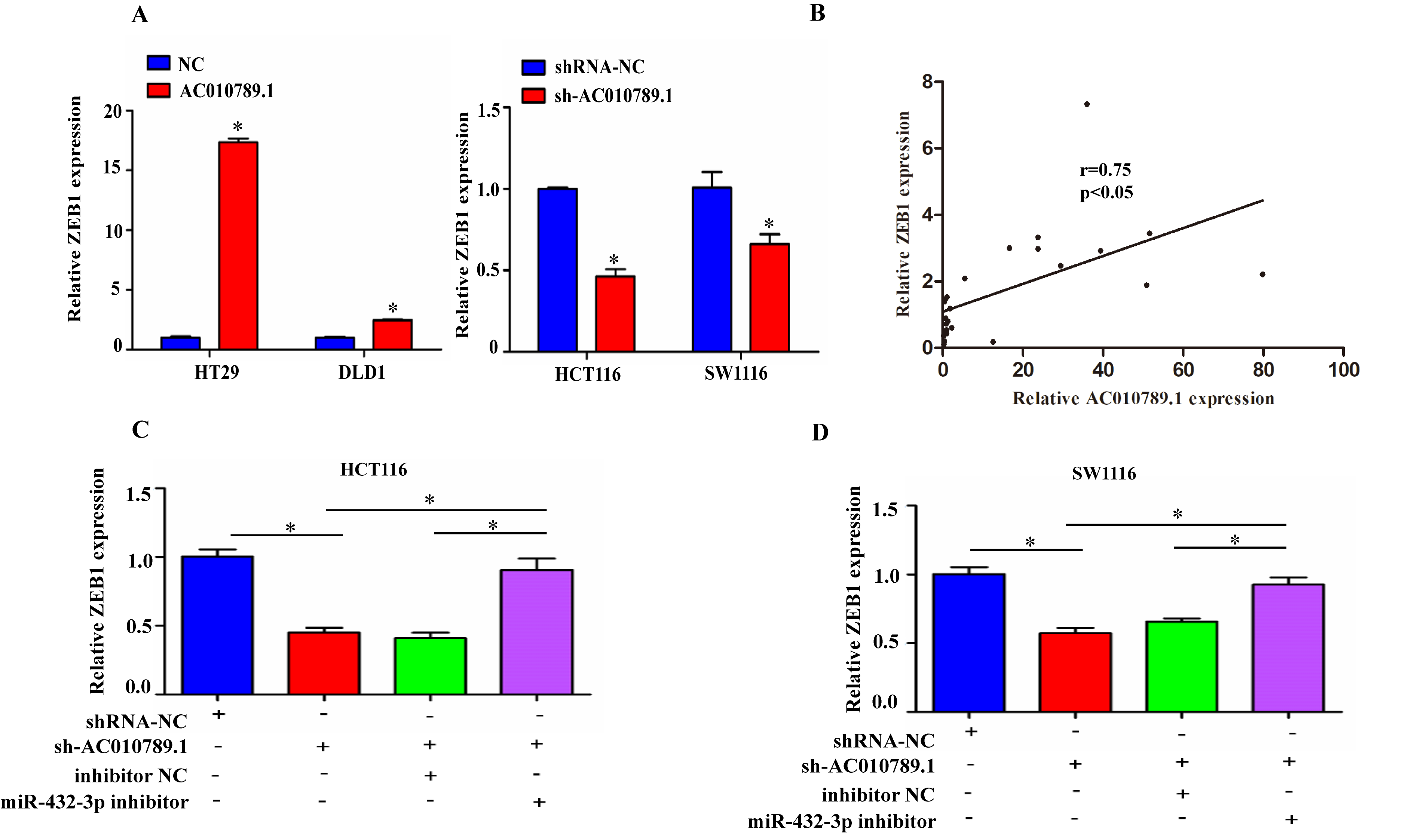

Supplement: Supplementary file 6 [file Image_5.TIF]

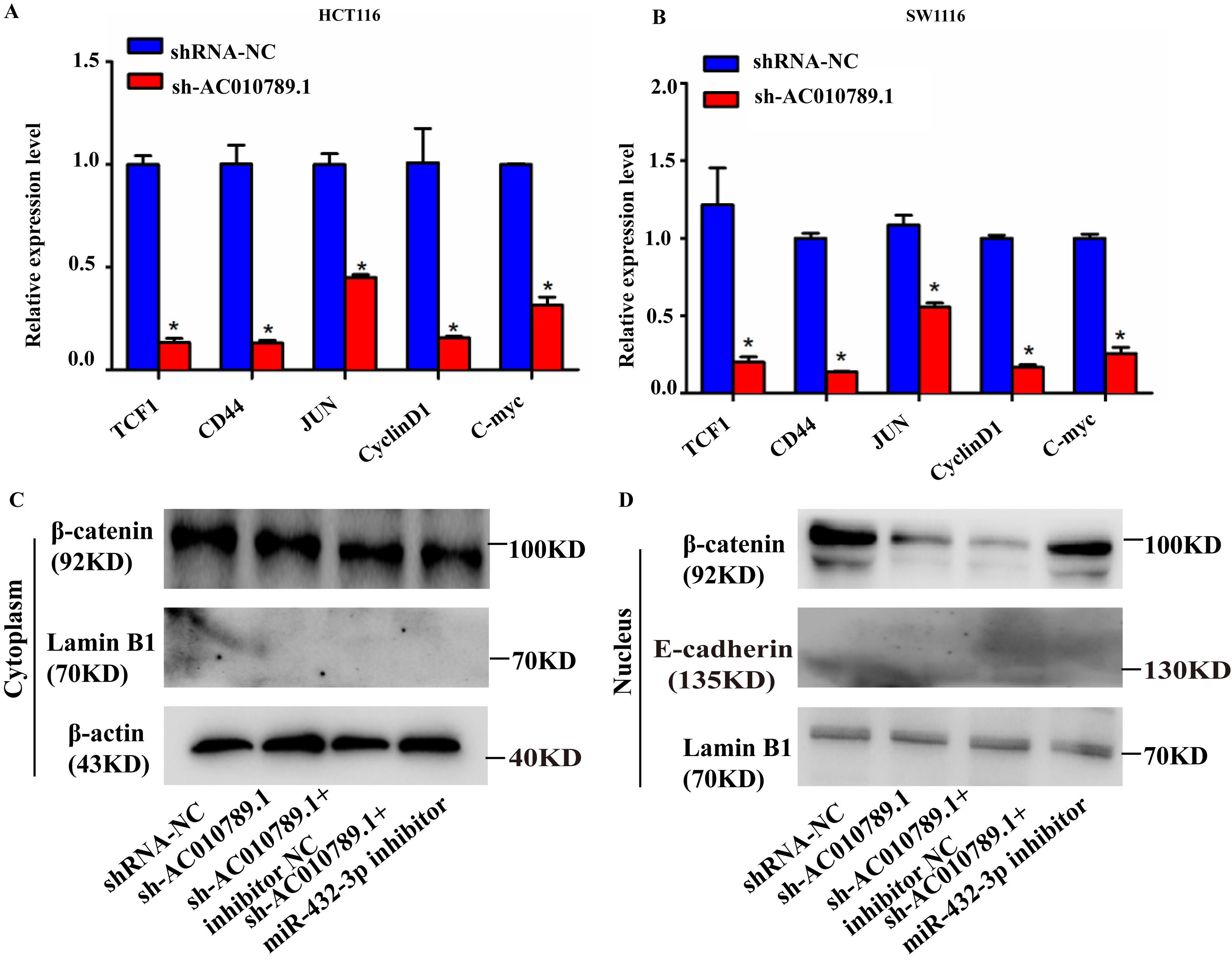

Supplement: Supplementary file 7 [file Image_6.TIF]
